# A Scalable Singlet Oxygen Reactor for Photodegradation of Active Pharmaceutical Compounds and Disinfection

**DOI:** 10.1021/acsestwater.5c00915

**Published:** 2026-03-06

**Authors:** Pabasara Samarawickrama, Hasanuwan Ihalagedara, QianFeng Xu, Christine Boisrobert, Rovshan Mahmudov, Alexander Greer, Alan M. Lyons

**Affiliations:** 1 Department of Chemistry, College of Staten Island, City University of New York, Staten Island, New York 10314, United States; 2 Ph.D. Program in Chemistry, The Graduate Center of the City University of New York, New York, New York 10016, United States; 3 SingletO2 Therapeutics LLC, 211 Warren St., Newark, New Jersey 07103, United States; 4 Air Liquide Innovation Campus Delaware, 200 Gbc Dr., Newark, Delaware 19702, United States; 5 Department of Chemistry, Brooklyn College, City University of New York, Brooklyn, New York 11210, United States

**Keywords:** polymer lightguides, photoreactor, reactive oxygen species, photooxidation, fluorinated phthalocyanines, APC photodegradation, bacterial inactivation, water treatment

## Abstract

Current water treatment approaches effectively inactivate pathogens; however, they are not designed to remove trace pharmaceuticals and other emerging contaminants. Singlet oxygen (^1^O_2_) offers a promising approach to water treatment due to its high oxidation potential while also being safe for humans and aquatic life due to its short lifetime (<4 μs in water) and rapid decay to molecular oxygen. However, this short lifetime makes implementation challenging. Moreover, obstacles to delivering light to the photosensitizer (PS) in turbid water must be overcome. Here, we present a novel reactor for generating ^1^O_2_ for water treatment. A water-insoluble PS is coated on the surfaces of roughened PMMA lightguides that are closely packed into a photoreactor and illuminated by a high-power LED. This approach is insensitive to water turbidity as the incident light is coupled to the PS coating. The effects of fluence, surface roughness, and PS loading on ^1^O_2_ trapping rates were quantified using uric acid. Destruction of five pharmaceutical compounds was measured by HPLC, and the rate constants demonstrate the influence of substrate structure on reactivity. Disinfection efficacy was demonstrated using 10^5^ CFU/mL *Escherichia coli* suspensions. Approaches to scaling this system for continuous water treatment are presented.

## Graphical abstract

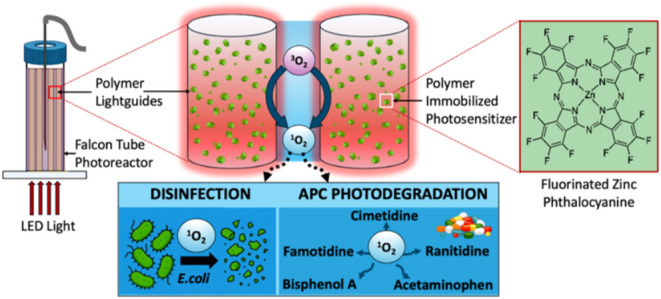

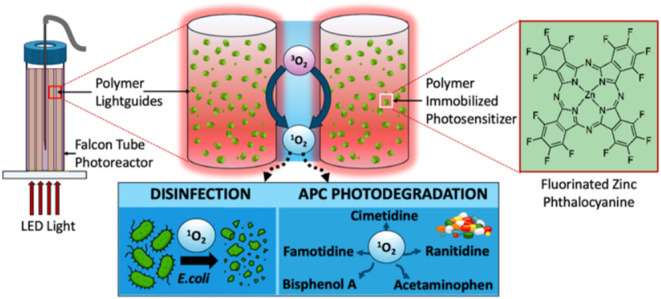

## 1 Introduction

Water pollution remains a critical challenge where contaminants such as active pharmaceutical compounds (APCs) and pathogenic microorganisms continue to threaten ecosystems and public health.^1,2^ APCs frequently contain functionalized aromatic groups making them resistant to oxidation, which enhances their environmental persistence.^3^ Some treatment methods, such as chlorination, are economical but can produce toxic and sometimes carcinogenic decomposition byproducts.^4^ Ozone is an effective treatment, but it is corrosive and poses a safety hazard to humans and aquatic life,^5^ requiring careful control of conditions for optimum use.^6^ Advanced oxidation processes (AOPs) generate a mixture of reactive oxygen species (ROS) that differ greatly in reagent selectivities. Moreover, AOP systems are energy intensive with high capital and operational costs that preclude their widespread use.^7,8^ UV light can be used to disinfect water but is adversely affected by turbidity. Suspended particles and dissolved organic matter (DOM) scatter and absorb light, which limits the penetration depth of incident light and the scalability of the UV reactors, especially for applications where turbidity is common, such as recirculating aquaculture systems (RAS).^9–12^ Thus, there is a need for developing a scalable reactor that can safely decompose APCs and inactivate pathogens, without exposing humans or aquatic life to toxic chemicals or carcinogenic byproducts. To this end, we have developed a scalable reactor for the production of a particular ROS, singlet oxygen (^1^O_2_), as a pure species.

Singlet oxygen is a valuable ROS with predictable mechanisms for effective APC decomposition. This ROS yields heteroatom oxidation and alkene and aromatic ring peroxidation.^13–15^ However, degradation rates are compound specific and may vary by several orders of magnitude. It also offers advantages, such as high oxidation potential and broad pH tolerance, while being safe for humans and aquatic applications as ^1^O_2_ rapidly decays to breathable oxygen in less than 1 ms in air and <3.5 μs in water.^16^ Despite these benefits, ^1^O_2_-based disinfection systems have not been extensively studied due to the challenges posed by the short lifetime of ^1^O_2_ in water. To address the need for a scaled-up system, we describe a photoreactor designed to overcome the limitations imposed by the short lifetime of ^1^O_2_ to efficiently remove APCs and inactivate microbes, paving the way for improved water treatment solutions.

Singlet oxygen arises when a photosensitizer (PS) absorbs light and transfers this energy to ground-state oxygen (^3^O_2_).^13^ Type I photosensitized oxidation gives rise to oxygen radical and radical ion intermediates.^17^ In contrast, type II photosensitized oxidation gives rise to singlet oxygen by energy transfer from an excited-state sensitizer to ground-state oxygen.^18^ Typically, the PS is dissolved in the solution to be treated, but such homogeneous systems are impractical for water treatment because the PS is difficult and expensive to recover and reuse. A solution to this problem is to immobilize the PS onto a solid support, thereby preventing PS release into the solution and creating a reusable system limited only by the stability of the PS.^19^ However, immobilizing PSs on solid supports (i.e., a heterogeneous system) introduces its own problems, namely, much lower ^1^O_2_ yields compared to homogeneous systems.^20^

PS-containing nanoparticles have the highest surface areas and fastest reactivities; however, they are especially difficult to isolate from the treated water and so their use poses environmental challenges.^21–23^ Particulate systems where the PS is coated onto micron-scale particle surfaces^16,24,25^ are difficult to illuminate uniformly as the large particles scatter and/or absorb incident light. Leaching of the PS into water, settling of the particles due to their density, and avoiding contamination of the water by particles that escape the reactor also pose obstacles to industrial applications.

Polymer-supported systems facilitate PS reuse and sequestration. In some approaches, PS molecules are embedded within a polymer matrix to avoid PS leaching into the surrounding water.^26–29^ When embedded, however, ground-state oxygen (^3^O_2_) must diffuse through the matrix before reacting with the PS; similarly, ^1^O_2_ generated from an embedded PS must diffuse through the matrix before it can react with an APC.^20,30^ This diffusion path reduces yields and limits PS to the outer few nm of the surface. Moreover, physical quenching of ^1^O_2_ by the matrix can also lower the overall yield.^19^ To address this challenge, our group has developed superhydrophobic (SH) surfaces that create high surface area supports for PSs. On SH supports, the PS is deposited selectively, adjacent to the solid–liquid–vapor interface where the vapor layer (plastron) acts as an oxygen reservoir thereby increasing yields 3× higher than on planar polymer substrates.^31^ However, coupling light efficiently to multiple layers of SH supports is challenging in part because the surfaces scatter light. Since the amount of ^1^O_2_ generated is linearly proportional to the absorbed light intensity,^32^ the optical properties of the PS support must also be carefully considered.

To efficiently couple light to supported PS, we hypothesized that yields of ^1^O_2_ will be increased by depositing small quantities of a photostable, water-insoluble PS onto light-emitting surfaces and that this approach will lead to the development of a scalable photoreactor that is cost-effective and energy efficient. To test this hypothesis, we developed an innovative reactor where a hydrophobic, insoluble, fluorinated phthalocyanine PS is deposited onto lossy polymer lightguide surfaces to increase ^1^O_2_ efficiency without the PS being leached into the aqueous solution.

A key aspect of this reactor is that the PMMA lightguide surface is roughened so that light entering the lightguide is scattered when it interacts with the surface, thereby illuminating the PS all along its length. Since incident light is transmitted through the lightguides, the system can operate equally well in clear water as well as in highly turbid environments. A second aspect of the optical design is to use red-light-emitting LEDs. Longer wavelength red light is less susceptible to scattering losses and absorption in turbid water.^33^ The energy efficiency of red LEDs (630–670 nm) is among the highest of any light source as these devices have been optimized for applications such as indoor agricultural and outdoor signage. Thus, their use paves the way to an energy-efficient water treatment method.

The PS used in this study, zinc 1,2,3,4,8,9,10,11,15,16,17,18,22,23,24,25-hexadecafluoro-29*H*,31*H*-phthalocyanine (ZnF_16_Pc), was selected due to its hydrophobic nature, insolubility, strong absorption in the red-light region (λ_max_ = 639 nm), and photochemical stability (i.e., resistance to photobleaching). The PMMA lightguide surfaces were coated with ZnF_16_Pc by immersion into an isopropyl alcohol solution of the PS. The PS-coated lightguides were tightly packed inside a 15 mm-i.d. polypropylene photoreactor bonded to a glass slide through which a red LED was coupled (Figure 1). We explored key parameters such as effect of fluence, surface roughness, and PS loading on ^1^O_2_ trapping rates, which were assessed using uric acid^34,35^ (UA) and furfuryl alcohol^36^ (FFA) trapping agents. The innovative reactor system was used to study the degradation of five different APCs that have been identified in surface waters and drinking-water supplies worldwide.^3,37–40^ The selection of these APCs has allowed us to access reactions of singlet oxygen with heterocycles and compounds with different functional groups, which enables an assessment of our photoreactor’s capabilities to degrade them. In parallel, we also assess the inactivation of *Escherichia coli* bacteria.

**Figure 1:**
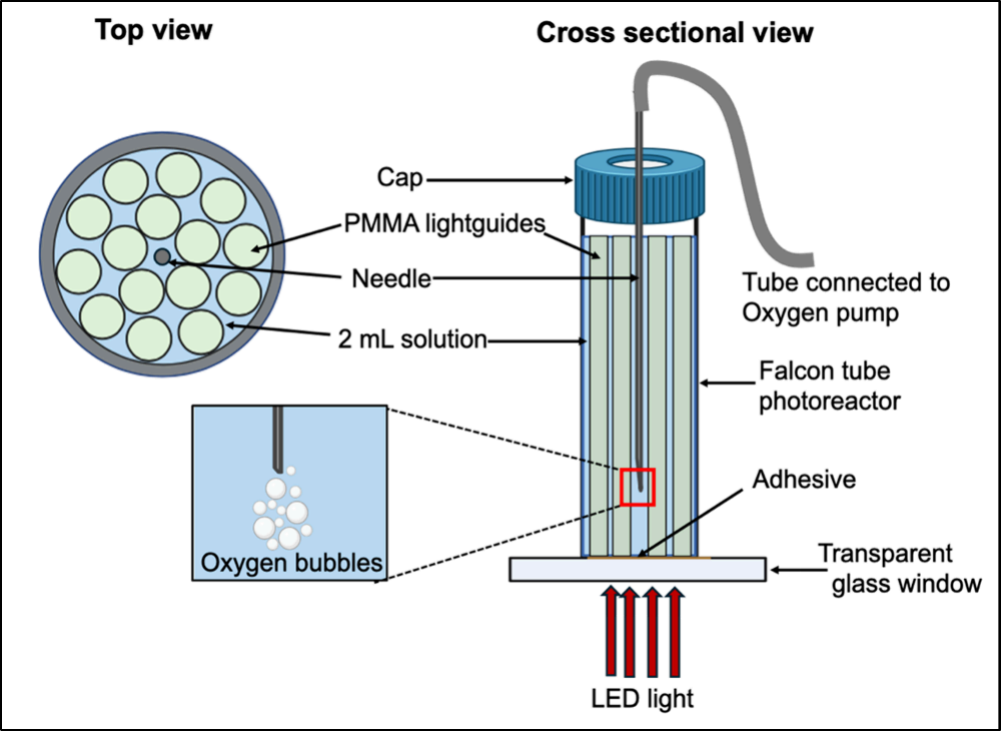
Schematic showing the top view and cross-sectional image of the photoreactor. Inset showing the oxygen bubbling into water through the syringe needle.

## 2 Materials and Methods

### 2.1 Chemicals and Reagents

Zinc 1,2,3,4,8,9,10,11,15,16,17,18,22,23,24,25-hexadecafluoro-29*H*,31*H*-phthalocyanine (ZnF_16_Pc, 90%), d-mannitol (≥99%), and 2-propanol (90%) were purchased from Sigma-Aldrich. Phosphate buffer saline (PBS) tablets were purchased from Medicago AB (Uppsala, Sweden). Anhydrous uric acid (UA, 99%), furfuryl alcohol (FFA, 98%), sodium azide (NaN_3_, ≥99%), and pharmaceutical compounds ranitidine hydrochloride (99%), famotidine (>97%), cimetidine (≥97.5%), bisphenol A (>97%), and acetaminophen (>98%) were purchased from Thermo Fisher Scientific. All chemicals were used as received.

### 2.2 Instrumentation

Each LED light source used a single Luxeon chip (Red LED with peak maxima at 637 nm, fwhm of 18 nm, and Green LED with peak maxima at 509 nm, fwhm of 27 nm) with a plain tight spot lens (Carclo) enclosed in 38.1 mm-diameter dynamic LED heat sink housing assembled by LEDSupply (Randolph, Vermont). The incident irradiance of the LED was measured by a visible-light-enhanced silicon photodetector (Model 918D-SL-OD3R, Newport Corp., Irvine, California), which was connected to the optical power meter (Model 1918-C, Newport Corp., Irvine, California). The desired irradiance of each LED was adjusted with a 20 kΩ terminal rotary potentiometer. The UV–vis spectral analysis of UA was performed using a PerkinElmer Lambda 650 UV–vis spectrophotometer. An oxygen concentrator (Hinor Medical POC-030) was used to supply oxygen for UA, APC, and bacterial experiments. A Nuvair Pro O_2_ hand-held analyzer was used to monitor the oxygen output from the oxygen concentrator. A 6″ diameter integrating sphere (StellarSphere IS6) was used to quantify the light transmission through the lightguides. Briefly, the PMMA lightguides coupled to LEDs were fully inserted through the 0° port of the integrating sphere (Section 3.3). The detector, SILVER-Nova spectrometer (StellarNet), was attached to the 90° port and analyzed using SpectraSuite software.

The photochemical degradation of pharmaceutical compounds was monitored by high-performance liquid chromatography (HPLC, Shimadzu Nexera X2 series). The APCs of cimetidine, ranitidine, famotidine, bisphenol A, and acetaminophen (10 μM each) were analyzed at 218, 319, 265, 225, and 245 nm detector wavelengths, respectively, at a constant temperature of 40 °C. The HPLC column (Raptor C18, 150 mm long × 3 mm i.d., 2.7 μm particle size) was used with a mobile phase of 90% DI water, 10% methanol, and 0.1% acetic acid with a flow rate of 0.5 mL/min. Bisphenol A (10 μM) was analyzed at 225 nm using a Pinnacle column (DB C18, 250 mm long × 3.2 mm i.d., 5 μm particle size) at a flow rate of 0.8 mL/min with 50% DI water and 50% methanol with 0.1% acetic acid. The number of significant figures reported for all measured and calculated values was determined based on the precision limits of the corresponding instruments. Measurement uncertainties and statistical errors are reflected as standard deviations where applicable.

## 3 Experimental Section

### 3.1 PMMA Lightguide Preparation and Coating

Twelve-inch-long PMMA lightguides (2.5 mm o.d. × 12″ long) were purchased from Vicenpal and mechanically roughened with sandpaper. The lightguides were rotated using a drill press while a piece of 180-grit sandpaper was applied to the surface, moving from top to bottom against the rotating PMMA. After roughening, the lightguides were cut into 4 cm lengths, and their faces were polished with an electric saw to enhance transparency. PS coatings on PMMA lightguides were done by dip-coating them in ZnF_16_Pc/IPA solutions for 15 min. After coating, the lightguides were thoroughly dried at room temperature at least 1 h. To wash off loosely bound PS, the lightguides were soaked in a 300 mL DI water bath for approximately 30 min, followed by rinsing with a continuous flow of DI water over the surfaces for approximately 5 min before the experiments. PS loading was quantified by solvent extraction followed by UV–vis spectroscopy. Roughened PMMA lightguides (*n* = 30) coated with 0.05 mg/mL PS solution were immersed in 5 mL of acetone and shaken vigorously for 30 s to desorb the PS. The acetone extract was analyzed by UV–vis using a Beer’s Law calibration curve (Figure S1), and loading was normalized to surface area as described in Table S1.

### 3.2 Photosensitizer Leaching Experiments

To test for PS leaching, 50 PMMA lightguides (coated using a 0.05 mg/mL PS solution) were soaked in 60 mL of PBS solution overnight. This leachate was analyzed for PS contamination using UV–vis spectroscopy, and no signal was observed at 639 nm, the peak of the ZnF_16_Pc absorption spectrum. The presence of PS in the leachate was further assessed based on ^1^O_2_ trapping using an 800 μM UA solution prepared from the leachate. A 2 mL aliquot of this solution was transferred into the photoreactor containing 15 PMMA lightguides with no PS coating and irradiated for 10 min using a red LED (100 mW/cm^2^ incident irradiance). If any PS was extracted into the leachate, a decrease in the UA signal would be expected. Changes in the UA concentration were monitored by measuring absorbance of UA at 291 nm by extracting 100 μL of the irradiated UA solution and diluting it with 900 μL of PBS. The experiment was repeated over five trials, and the results show no evidence of ^1^O_2_ generation (Figure S2).

### 3.3 Light Transmission through Lightguides

The transmission of light through the lightguides was studied by coupling LED light to smooth and roughened PMMA lightguides, which were inserted into an integrating sphere, as shown in Figure S3a. To measure the light emitted from the 4 cm length used in the photoreactor, 6 cm lightguides were used such that only 4 cm-long segments were exposed within the integrating sphere. A portion of the lightguide was outside the sphere to enable coupling to the light source. To quantify sidewall scattering, the tip was capped (covering only the last 1–2 mm of the lightguide) to block the polished end-face emission while ensuring that emission was recorded from the same 4 cm length in both capped and uncapped configurations. Similarly, the total light intensity emitted by the lightguide was measured by introducing uncapped lightguides into the integrating sphere (Figure S3b). The light transmitted through the polished end was calculated by subtracting the sidewall emission value from the total emission. The LED light sources were chosen to have either the maximum overlap with the ZnF_16_Pc absorption spectra (red LED) or a minimal overlap with ZnF_16_Pc absorption (green LED) to quantify the percentage of light absorbed and scattered by the PS present on the sidewalls of the lightguides, respectively (Figure S4).

### 3.4 Photochemical Experiments with Uric Acid

The photochemical experiments were carried out by packing 15 of the PS-coated PMMA lightguides into a photoreactor fabricated from a 15 mL Falcon tube (15 mm i.d. and 60 mm tall) with the tapered bottom of the tube cut away and a glass window adhered in its place, as shown in Figure 1.

Uric acid (800 μM) in pH 7.4 phosphate buffer (PBS) was used as the ^1^O_2_ trapping solution and stored in an ice bath in the dark prior to use. Experiments were performed at a temperature of 18–20 °C. The photoreactor was filled with 2 mL of UA solution to cover the 4 cm-long PMMA lightguides. Oxygen was bubbled into the solution using a needle inserted through the reactor cap and connected to the oxygen concentrator that supplied ∼90% oxygen. The incident irradiance of the red LED was maintained at 100 mW/cm^2^ as measured using a visible-light-enhanced silicon photodetector. During photochemical experiments, the system was irradiated for 10 min, while absorbance measurements were obtained by opening the cap to collect 100 μL UA aliquots using a pipet at time intervals of 0, 4, 6, 8, and 10 min. The amount of ^1^O_2_ trapped was determined by monitoring the decrease of absorbance peak for UA at 291 nm using UV–vis spectroscopy. Aliquots of 100 μL collected for each measurement were diluted with 900 μL of phosphate-buffered saline (PBS) for analysis using disposable semimicro PMMA cuvettes (BrandTech, 759075D, 1.5 mL volume capacity and 1 cm path length). The trapping rate of ^1^O_2_ (mmol•h^–1^ L^–1^) was calculated using the UA degradation rate using the method shown in Table S2.

### 3.5 Photochemical Experiments with APCs

The photochemical degradation of pharmaceutical compounds, famotidine, ranitidine, cimetidine, bisphenol A, and acetaminophen were carried out by preparing 100 μM solutions in aqueous PBS solutions at room temperature using the same reactor as for the UA experiments described above. A volume of 2 mL of the solution was added to the photoreactor and irradiated at 300 mW/cm^2^ with a red LED. For reaction rate calculations, a standard ^1^O_2_ probe, furfuryl alcohol (FFA, 100 μM), was used; irradiation was done under the same conditions as for the APCs. For each reaction, 100 μL samples were removed periodically for HPLC analysis. The total irradiation time and sampling intervals varied depending on the compound, with six data points collected for each trial. For famotidine, ranitidine, and cimetidine, the total irradiation period was 300 s, with samples collected every 60 s. For bisphenol A and acetaminophen, the total irradiation time was 1500 s, with samples taken every 300 s. At least three trials were performed for each APC and FFA. After irradiation, each APC solution and FFA were diluted to 10 μM for HPLC analysis and kept refrigerated in the dark until analyzed. A plot of ln­[FFA] vs time is shown in Figure S5. Direct photolysis controls (no PS) were conducted under conditions identical to those used for the APCs. The results, shown in Figure S6, demonstrate that the solutions are stable when irradiated only with light (no PS coating) with concentrations reduced by <2% after 480 s of irradiation.

To verify that singlet oxygen was responsible for APC degradation, solutions of sodium azide, a ^1^O_2_ chemical quencher, and d-mannitol, a superoxide anion and hydroxyl radical quencher, were used. Either 5 mL of a 4 mM solution of sodium azide or 5 mL of a 400 μM d-mannitol solution was added to 5 mL of a 200 μM solutions of FFA, cimetidine, or a 1:1 mixture of FFA–cimetidine (200 μM each). A volume of 2 mL of a solution was added into a clean photoreactor and irradiated for 10 min with red LED at 300 mW/cm^2^ incident irradiance. Aliquots of 100 μL were collected after each irradiation period and were diluted to 10 μM using PBS for HPLC analysis. The solutions were refrigerated in the dark until analyzed. Plots of ln *C*/*C*_o_ vs time are shown for cimetidine and FFA in Figure S7. Rates of cimetidine and FFA degradation with and without inhibitors, are shown in Table S3.

### 3.6 Chemical Reaction Rate Constant (*k*_r_) with ^1^O_2_

The chemical reaction rate constant (*k*_r_) of APCs and FFA with ^1^O_2_ was calculated using eq 1.^37^ The value of *k*_r_ is equal to the ratio of the slopes of the first-order plots of each compound (ln­(*C*/*C*_o_) vs time) and FFA (ln­(FFA/FFA_o_) vs time) multiplied by the value of *k*_r_ for FFA, where *C* is the concentration at time (*t*) and *C*_o_ is the initial concentration of each compound.

$$k_{r}^{compound}=\frac{\ln\left(\frac{C}{Co}\right)}{\ln\left(\frac{FFA}{{FFA}_{0}}\right)}.k_{r}^{FFA}$$ 1

Furfuryl alcohol was used as the reference molecule because it rapidly reacts with ^1^O_2_ and the reaction rate constant value for FFA (*k*_r_^FFA^) is reported to be 1.0 × 10^8^ M^–1^ s^–1^.^36^ The steady-state concentration of ^1^O_2_ was calculated as described previously,^41^ assuming a first-order decay of FFA shown in Figure S5 and Table S4.

### 3.7 Bacterial Disinfection Study

*Escherichia coli* (ATCC 25922) was selected as the target organism for disinfection studies. To prepare bacterial suspensions, a sterile solution of Luria–Bertani (LB) broth was inoculated with *E. coli* taken from an active culture and incubated at 37 °C for approximately 3–4 h to achieve a bacteria population in the stationary/log phase. The culture was centrifuged to prepare a bacteria pellet, followed by removing the growth media, washing with PBS, and suspending the pellet in sterile PBS. Bacterial suspensions of 1 × 10^5^ CFU/mL were prepared in PBS, and disinfection studies were conducted with an incident irradiance of 550 mW/cm^2^ for 2 h for a total fluence of 3980 J using PMMA lightguides coated with a PS concentration of 0.05 mg/mL. The bacterial suspensions were maintained at temperatures between 24 and 28 °C throughout the experiment. Oxygen (∼90%) was continuously bubbled into the solution using a needle inserted through the reactor cap. Bacterial inactivation efficiency was assessed by extracting samples (50 μL) at different time intervals (15, 30, 60, 90, and 120 min). Serial dilutions in sterile PBS were plated on Luria agar (100 μL) and incubated at 37 °C for ∼15 h, followed by colony counting.

## 4 Results

### 4.1 Effect of Surface Roughness on Light Delivery

#### 4.1.1 Effect of Sidewall Roughening on Light Delivery

The effect of surface roughness on light delivery was studied using both smooth and roughened PMMA lightguides coupled to red light. A smooth lightguide effectively transmits light from one end to the other with little light leaking out the sidewalls. This is demonstrated by comparing the maximum peak emission intensity (λ_max_ = 639 nm) from the lightguide before and after blocking the far end of the lightguide, as shown in Figure 2. According to the light intensity measurements, a smooth lightguide with an end cap showed a 94 ± 0.4% reduction in incident light intensity relative to an uncapped waveguide (Figure 2a), whereas roughened lightguides showed only a 5.1 ± 0.6% drop when capped (Figure 2b). These results indicate that smooth lightguides deliver 94% of the light through the polished end, scattering only ∼6% through the sidewalls. In contrast, roughened lightguides scatter the majority of incident light (95%) through the sidewalls, with only ∼5% transmitted through the polished end (Table S5).

**Figure 2:**
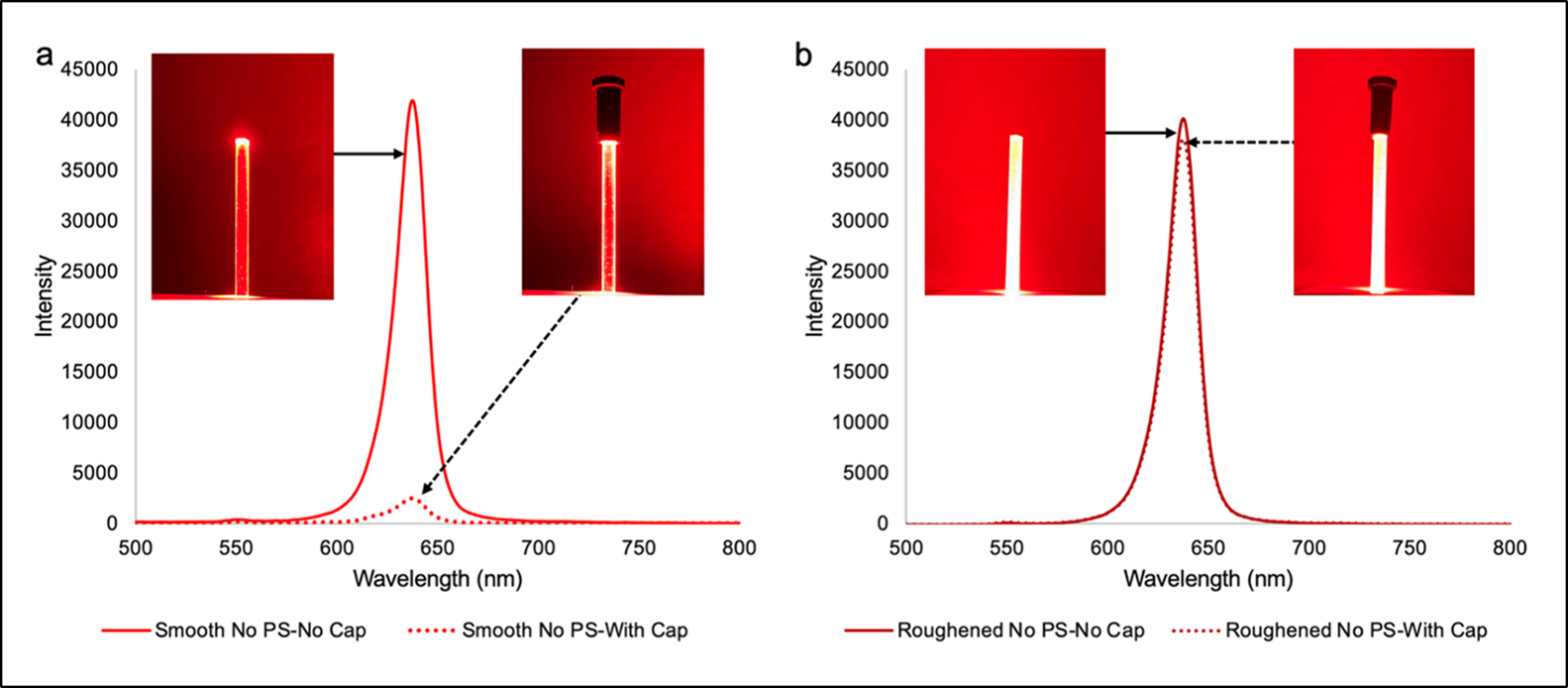
Diffuse emission spectra and photographs illustrating the relative intensity of light scattered along the lightguide sidewalls vs transmitting through the lightguide for uncapped and capped lightguides coupled to a red LED with sidewalls that are (a) smooth and (b) roughened.

#### 4.1.2 Effect of PS on Light Delivery through the Lightguides

To study the effect of PS particles on light delivery through the PMMA lightguides, red and green LEDs were coupled to PS-coated lightguides to assess the absorbance and scattering, respectively. Measurements were done using both smooth and roughened lightguides, with and without the end caps.

The PS coating absorbed red light that was coupled into the lightguide from the LED, thereby reducing the intensity of light emitted from the sidewalls and polished end, as shown in Figure S8 and Table S5. On smooth lightguides, the reduction was 29 ± 6% compared to smooth lightguides without PS, whereas on roughened lightguides, the emitted light was reduced by 25 ± 1% relative to lightguides with no PS. This indicates that the PS coated on sidewalls absorbs red light similarly regardless of surface roughness.

In contrast, when a green LED was coupled to the lightguides, the presence of PS reduced the light emission by a smaller amount for both smooth (5.4 ± 4.0%, Figure S9a) and roughened (2.3 ± 4.1%, Figure S9b) lightguides; results for total emission and sidewall emission are summarized in Table S6. This demonstrates that the PS absorbs red light from within the lightguide to a much greater extent than green light (data summarized in Table S7), which is consistent with the complete overlap of the red LED emission band with the absorption band of the PS and the minimal overlap of the green LED emission band with the PS shown in Figure S4.

### 4.2 Effect of Surface Roughness on the ^1^O_2_ Trapping Rate

The rates of ^1^O_2_ trapping from both smooth and roughened lightguides in the photoreactor are shown in Figure 3. The 291 nm UA peak exhibited a linear decrease (*R* ^2^ > 0.99) over time for both types of lightguides, with UA concentrations decreasing by 44% (Figure 3, blue trace) and 65% (Figure 3, red trace) for smooth and roughened lightguides, respectively, after 600 s (10 min) of illumination. This corresponds to rates of 2.12 and 3.13 mmol•h^–1^ L^–1^—a 48% faster trapping rate for the roughened lightguides. Although the reaction of UA with ^1^O_2_ is bimolecular, the plot of UA absorbance vs time is linear because of the >10^6^ higher concentration of UA (800 μM) compared to the steady-state concentration ^1^O_2_ (3.3 × 10^–11^ M).

**Figure 3:**
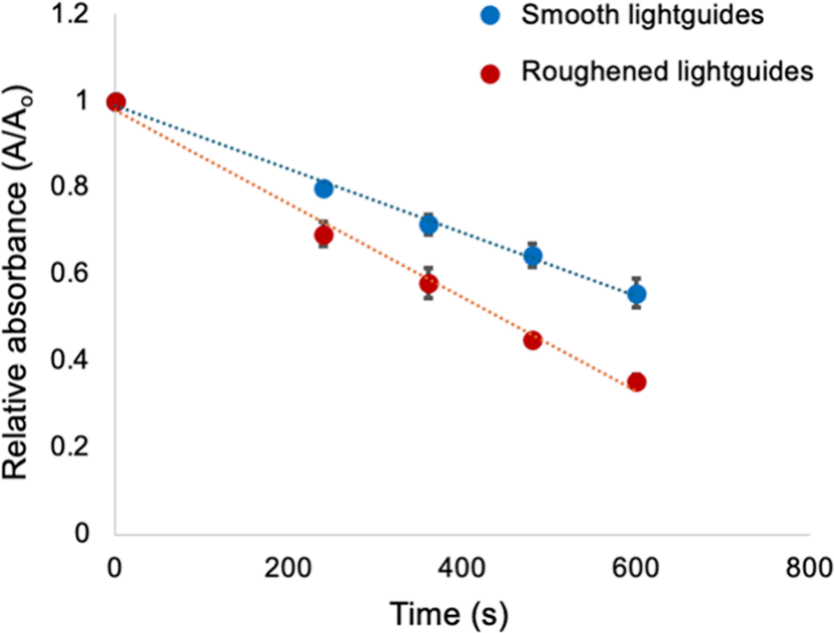
Plots of absorbance at 291 nm (UA absorbance peak) vs time for smooth (blue trace) and roughened (red trace) lightguides used to calculate relative rates of ^1^O_2_ production. Error bars represent the standard deviation for at least three different trials.

### 4.3 Effect of PS Loading on the ^1^O_2_ Trapping Rate

To study the effect of PS loading on the ^1^O_2_ trapping rate, the PMMA lightguides were coated using a range of PS solution concentrations. As the PS solution concentration increased, the quantity of PS deposited on the lightguide surface increased as shown by the decreasing relative transmittance (%*T*) measured in the integrating sphere using LED illumination at 639 nm, the peak of the ZnF_16_Pc absorbance (Figure 4, blue trace). %*T* decreased linearly up to a concentration of 0.05 mg/mL. At higher concentrations, the transmittance change slowed, and the variance between samples increased, indicating that the PS crystallite coating on the PMMA surfaces approached saturation.

**Figure 4:**
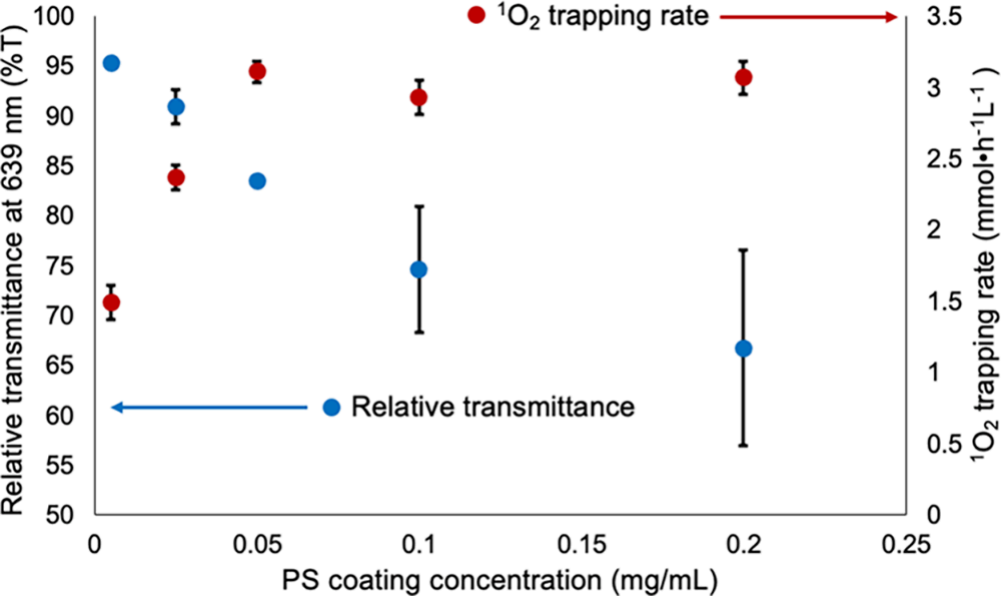
Plots showing the relative %*T* of ZnF_16_Pc-coated lightguides at 639 nm as a function of PS concentration (blue trace) and corresponding ^1^O_2_ trapping rate at each coating concentration (red trace). The relative %*T* represents light transmitted through PS-coated lightguides normalized to that of lightguides with no PS coating. All the %*T* measurements were collected from roughened lightguides without a cap. Error bars represent the standard deviation for at least three different trials.

Increasing the PS loading level on the PMMA surface resulted in a linear increase in the amount of ^1^O_2_ trapped up to a concentration of 0.05 mg/mL, as shown by the red trace in Figure 4. Above this coating solution concentration, however, the rate of ^1^O_2_ trapped remained constant, showing no significant increase. The loading of ZnF_16_Pc coated on the surface of a roughened PMMA lightguide using a PS solution with a concentration of 0.05 mg/mL was 7.1 × 10^–4^ mg/cm^2^, as determined by UV–vis spectroscopy as described in Figure S1 and Table S1.

### 4.4 Photochemical Degradation of Pharmaceutical Compounds

To study the photochemical degradation of APCs, the compounds cimetidine, ranitidine, famotidine, bisphenol A, and acetaminophen were evaluated with the photoreactor system. First-order plots (ln­(*C*/*C*_o_) vs time) are shown in Figure 5. All the compounds show a linear first-order behavior with *R* ^2^ ≥ 0.95. Cimetidine shows the fastest degradation rate of 8.8 × 10^–3^ s^–1^ with approximately 92% of the compound degraded within 5 min of irradiation. Both ranitidine and famotidine show relatively slower degradation rates of 2.5 × 10^–3^ and 2.3 × 10^–3^ s^–1^, respectively, with 53 and 50% degradation within 5 min. Bisphenol A and acetaminophen are more resistant to degradation, with rates of 3.1 × 10^–4^ and 4.6 × 10^–5^ s^–1^, respectively, with 37 and 5% removal after 25 min of irradiation.

**Figure 5:**
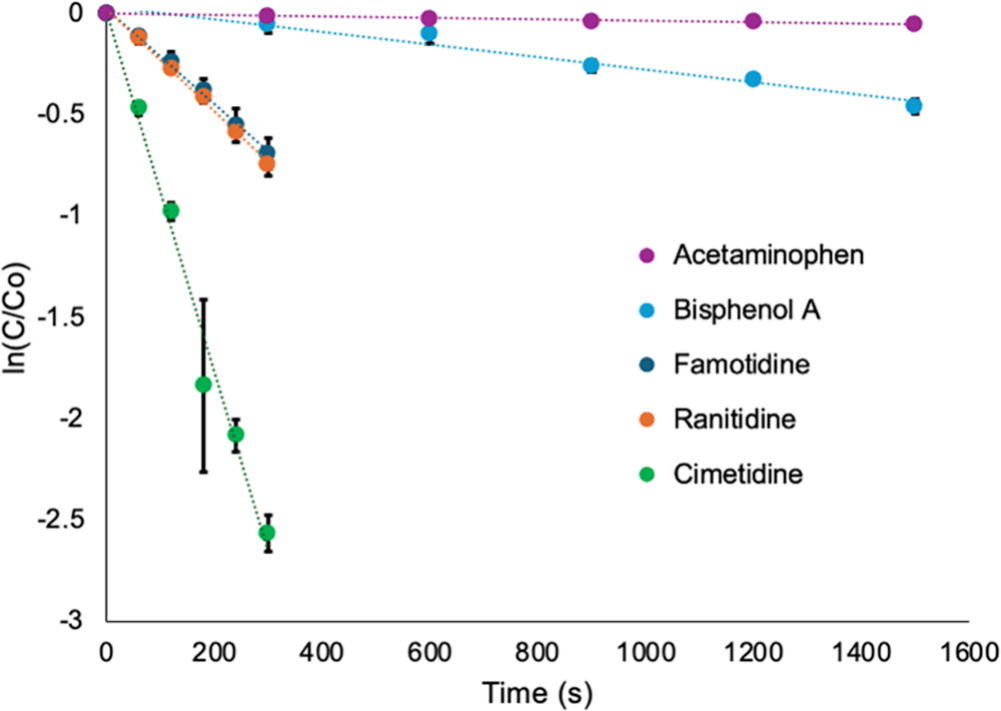
First-order plots of pharmaceutical compound degradation as a function of irradiation time. The *R* ^2^ values obtained from the first-order plots were as follows: acetaminophen (*R* ^2^ = 0.95), bisphenol A (*R* ^2^ = 0.97), famotidine (*R* ^2^ = 0.99), ranitidine (*R* ^2^ = 0.99), and cimetidine (*R* ^2^ = 0.98). Error bars represent the standard deviation for at least three different trials.

The *k*_r_ values for the reaction between ^1^O_2_ and each compound, shown in Table 1, were obtained using FFA as the reference compound as described by eq 1 (Section 3.6). The steady-state concentration of ^1^O_2_ was calculated as described previously^41^ using the equation *k*_obs_/*k*_r_ = [^1^O_2_]_ss_, where *k*_obs_ is the slope of ln­[FFA] vs time (Figure S5 and Table S4) and *k*_r_ is the reaction rate constant from the literature of (1.0 × 10^8^ M^–1^ s^–1^);^36^ [^1^O_2_]_ss_ was then calculated to be 3.3 × 10^–11^ M.

**Table 1:**
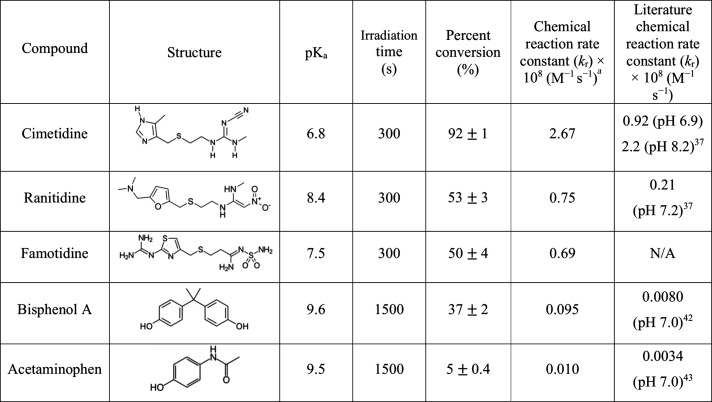
Summary of Irradiation Time, Percent Conversions, and Chemical Reaction Rate Constants of ^1^O_2_ ^37,42,43^ ^b^

a pH was buffered at 7.4.

b The rate constants (*k*_r_) for each compound were obtained from the best-fit linear regression lines of the corresponding first-order plots. Standard deviation values were obtained from at least three independent trials

Cimetidine exhibits the highest *k*_r_ value, which is 3.7 times higher than that of famotidine and ranitidine, 28 times higher than BPA, and 270 times higher than acetaminophen.

### 4.5 Effect of ROS Quenchers, Sodium Azide (NaN_3_), and d-Mannitol, on *k*_r_

To demonstrate that ^1^O_2_ is the ROS responsible for pharmaceutical compound degradation, NaN_3_ a ^1^O_2_ quencher, and d-mannitol, a superoxide anion and hydroxyl radical quencher were added to solutions containing a mixture of cimetidine and FFA (100 μM each), followed by irradiation in the photoreactor (concentrations of NaN_3_ and d-mannitol after addition to FFA were 2 mM and 200 μM, respectively*)*. First-order plots for cimetidine and FFA (when mixed in a 1:1 ratio) are shown in Figure 6.

**Figure 6:**
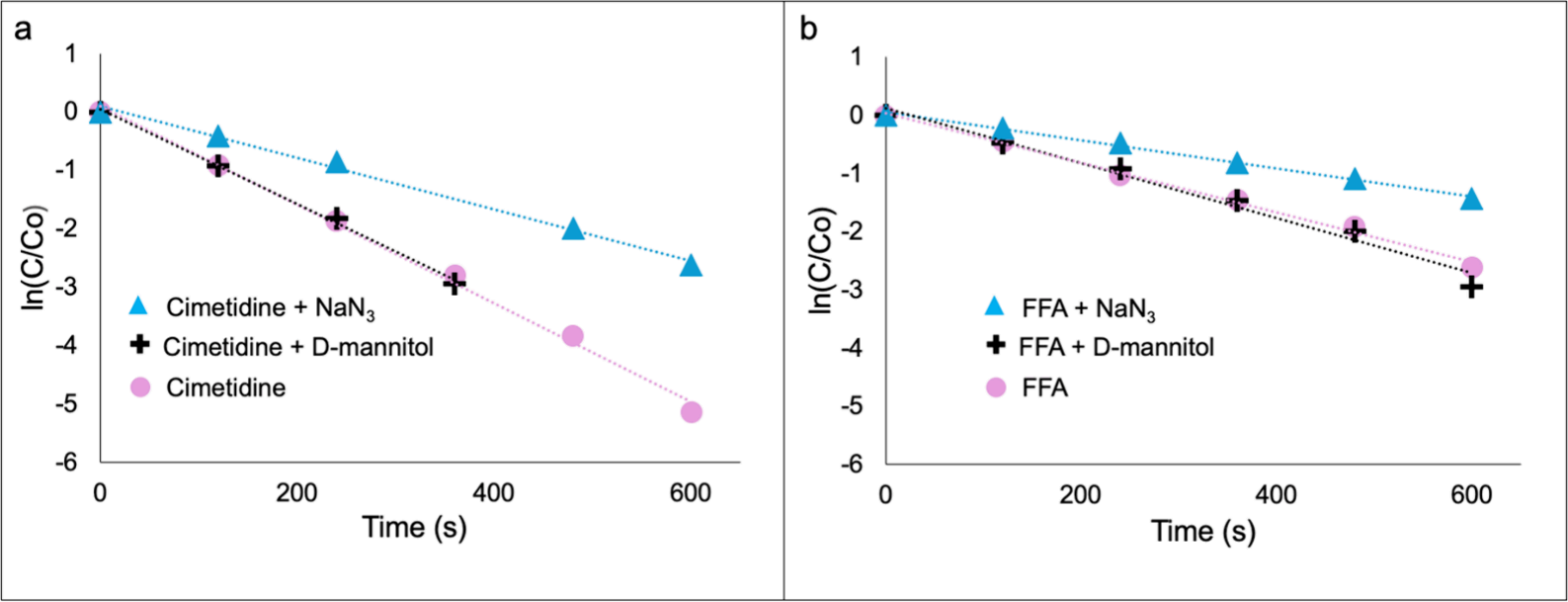
First-order plots of (a) cimetidine degradation and (b) FFA degradation with the addition of d-mannitol and NaN_3_. Each plot exhibited an *R* ^2^ value >0.99.

Degradation rates of a mixture of cimetidine (Figure 6a) and FFA (Figure 6b) were not affected by the addition of d-mannitol, suggesting that type I photochemistry plays little or no role in the degradation of the APCs compared to singlet oxygen. However, adding NaN_3_ reduced degradation rates of cimetidine and FFA by 45 and 41%, respectively. When cimetidine and FFA were evaluated independently using 100 μM solutions, a similar trend was obtained as shown in Figure S7. Degradation rates were not affected by d-mannitol while the degradation rates were reduced by 61 and 43%, for cimetidine and FFA, respectively, with addition of NaN_3_.

### 4.6 Bacterial Study

Bacterial inactivation efficacy increased linearly (*R* ^2^ > 0.95) as a function of the incident light dose, as shown in Figure 7 for an initial concentration of 10^5^ CFU/mL of *E. coli*. A 90% bacterial reduction (1 log) was achieved after 2 h of irradiation, resulting in an inactivation efficiency of 2.15 × 10^–2^ %/J. Control experiments carried out without PS also led to a reduction in CFU levels, but at a slower rate such that a 47% reduction was observed after 2 h under red LED illumination (Figure 7 green points). A similar reduction was observed using lightguides without PS and without LED illumination (Figure S11). This reduction of bacterial concentrations is consistent with adsorption of bacteria onto the roughened, exposed, PMMA surfaces.^44^ When lightguides are coated with PS, the surfaces become hydrophobic and a minimal impact on bacterial concentrations was observed.

**Figure 7:**
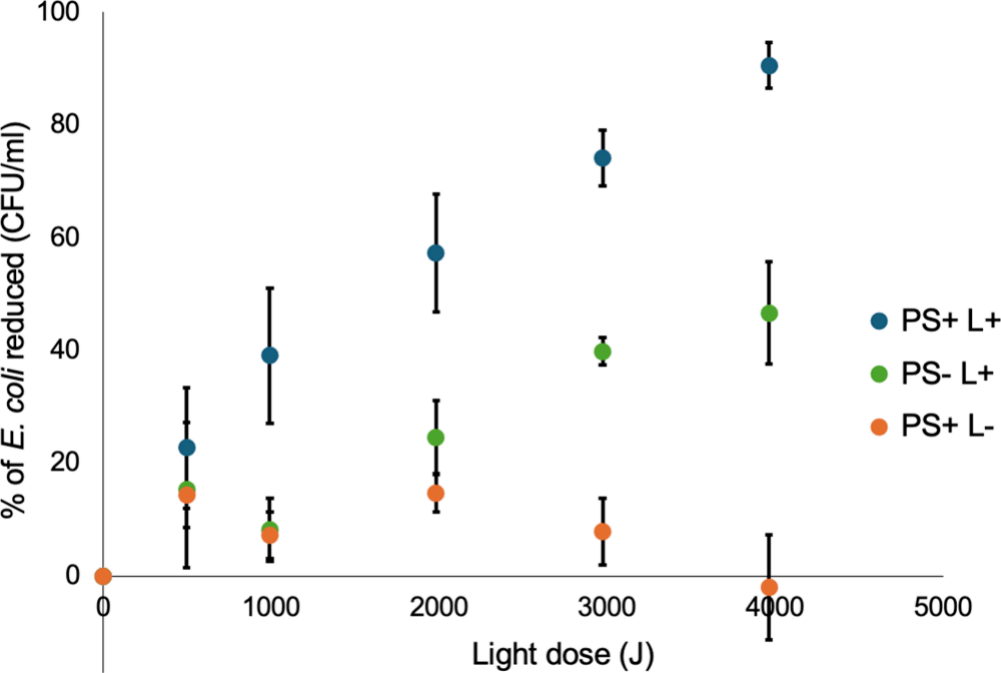
Effect of light dose on the reduction of bacterial CFU levels: blue trace, PS with light; green trace, no PS with light; and orange trace, PS without light. The error bars represent standard deviations from at least three independent trials.

## 5 Discussion

### 5.1 Factors Affecting ^1^O_2_ Generation

This study highlights the effectiveness of generating ^1^O_2_ using a photoreactor where the PS is supported on PMMA lightguides. The effectiveness of the system is further demonstrated by the relatively high steady-state ^1^O_2_ concentration achieved with the PMMA lightguide system (3.3 × 10^–11^ M), which is greater than values reported for our previously reported heterogeneous systems, including fiber optic ^1^O_2_ generator device (1 × 10^–14^ M),^45^ three-phase superhydrophobic sandwich (1.8 × 10^–13^ M),^41^ and two-phase SH systems (7.0–8.5 × 10^–12^ M).^46^ DOM in natural waters can also generate ^1^O_2_ under solar irradiation; however, reported steady-state concentrations of ^1^O_2_ in DOM systems (measured using FFA as a probe) are typically in the range of 10^–15^–10^–13^ M,^47–49^ which are two to four orders of magnitude lower than in our PMMA lightguide system.

An important factor contributing to the efficacy of the photoreactor is the light distribution system and its efficient illumination of the PS coating. Illumination of the PS coating occurs by two pathways: light coupled into the bottom of the lightguide (∼57%) and light passing through the gaps between lightguides (∼43%), as illustrated schematically in Figure 8. Light coupled into the lightguide can be absorbed by the PS coating, as the light encounters the PMMA surface. The percent of light absorbed by the PS is similar on smooth (29%) and roughened (25%) lightguides. Scattering at the PMMA surface, however, is highly dependent on roughness (Figure 2); the smooth lightguides scatter 6% of the light from the sidewalls whereas roughened lightguides scatter 71% of the light that enters the lightguide. This scattered light illuminates the PS coated on neighboring lightguides and thus accounts, in large part, for the 48% greater ^1^O_2_ output of roughened lightguides compared to smooth lightguides, as shown in Figure 3.

**Figure 8:**
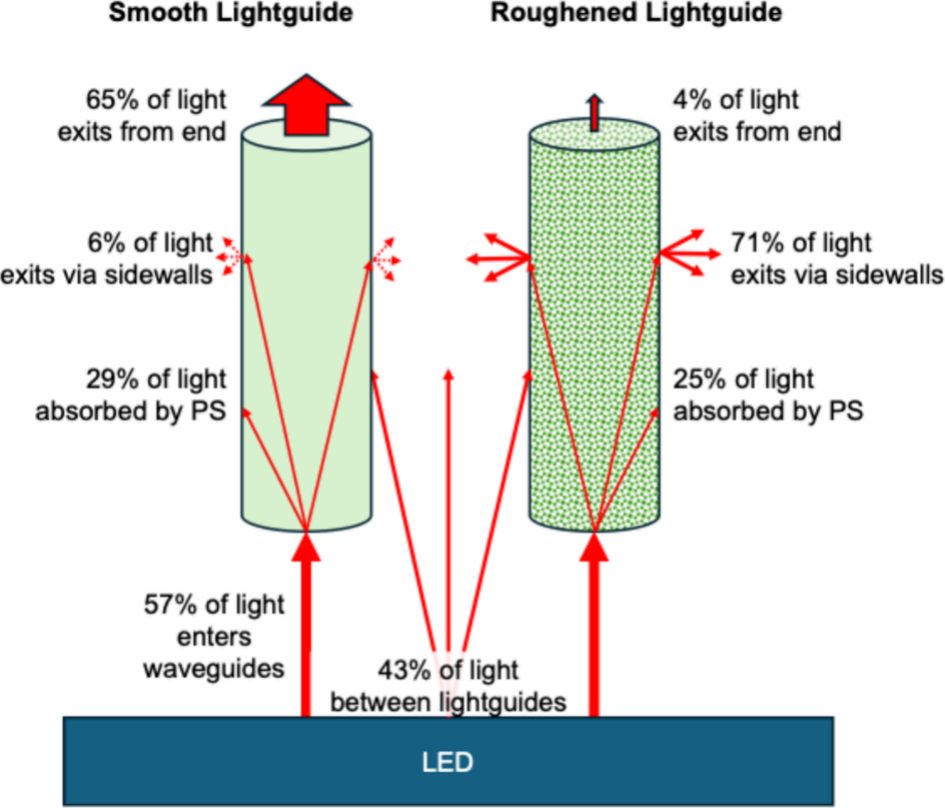
Schematic illustrating the major light pathways for smooth and roughened lightguides in the photoreactor.

Thus, much of the light that enters the smooth lightguides exits through the top (65%) and is wasted, lowering system efficiency whereas only 4% of the light exits the top of roughened lightguides. As these measurements were conducted in air, the amount of light scattered when the lightguides are immersed in water would be expected to be greater than when measured in air, as predicted by Snell’s law due to the higher index of refraction of water (1.33) vs air (1.00).

It is interesting to note that the PS coating does not enhance emission from the sidewalls. When illuminating the lightguides with the green LED (with a wavelength that is not absorbed by the PS), the PS coating results in a relatively small decrease of the total emitted light for both smooth (−5.4%) and rough (−2.3%) lightguide surfaces, as shown in Figure S9 and Table S7. Even though light is not scattered by the PS particles, the PS coating absorbs significant amounts of red light (29% on smooth surfaces and 25% on rough surfaces).

The other important factor that affects ^1^O_2_ generation is the PS loading on the lightguide surface. Increasing PS coverage leads to higher ^1^O_2_ trapping rates. However, once the majority of the surface is covered, additional PS deposition does not further increase rates. Coating with a PS concentration of 0.05 mg/mL (approximately 7.1 × 10^–4^ mg/cm^2^ loading) exhibited the highest ^1^O_2_ trapping rate of 3.13 mmol•h^–1^ L^–1^ (Figure 4). Previous studies showed a similar trend and correlated this effect with the size of PS crystallites on planar surfaces by SEM.^31^ Excess PS can be detrimental as the PS within large crystallites, or otherwise unavailable to the solution, will absorb light, which increases the temperature of the PS, without generating ^1^O_2_. Higher PS temperatures would accelerate PS degradation mechanisms.

### 5.2 APC Photodegradation

The photoreactor was shown to decompose five different APCs; however, the degradation rate depends on the chemical structure of the APCs. This difference arises from the selective ability of ^1^O_2_ to oxidize compounds; ^1^O_2_ reacts preferentially with electron-rich unsaturated organic compounds, in contrast to the less-selective oxidation by hydroxyl radicals (·OH).^42^ APCs such as cimetidine degraded rapidly, 92% after 5 min of irradiation, whereas acetaminophen was degraded by only a few percent in that time. The addition of NaN_3_, a selective ^1^O_2_ quencher, inhibited the degradation of cimetidine and FFA by 41–45%, although the extent of inhibition may vary with quencher concentration.^50^ In contrast, a common type I trapping compound (d-mannitol) showed no inhibitory effect, indicating that the reaction proceeds mainly by ^1^O_2_ via a type II pathway.^51^

The reactivity of APCs with ^1^O_2_ is due to the presence of heterocycles and nucleophilic groups. The higher reactivity of cimetidine, ranitidine, and famotidine is attributed to their imidazole, furan, and thiazole rings that can form endoperoxides from [2 + 4] cycloadditions, as well as their sulfide sites that can form sulfoxides and sulfones. Both sites can react with ^1^O_2_; such tandem reactions have been reported.^52–54^ Many ^1^O_2_ reactions with heterocycles and sulfides have been reported individually, but these reports have not often focused on these reactions for APCs.

Comparisons of ^1^O_2_’s chemical reactivity to segments of the APCs are useful for gaining insight into the faster reactivity of cimetidine, ranitidine, and famotidine relative to bisphenol A and acetaminophen. For example, imidazole compounds have *k*_r_ values mainly ranging from 1 to 3 × 10^7^ M^–1^ s^–1^,^55,56^ and furan compounds often ranging from 5 to 10 × 10^7^ M^–1^ s^–1^.^36,57^ *k*_r_ values for thiazoles (a structural unit in famotidine) have not been previously reported^57^ but would be expected to be similar to imidazoles and furans. Diethylsulfide chemically reacts with ^1^O_2_ at a similarly rapid rate (*k*_r_ = (3–4) × 10^7^ M^–1^ s^–1^).^58^ Heterocycles and sulfide groups are electron rich and thus react rapidly with ^1^O_2_. In contrast, phenol compounds, similar to acetaminophen, react ∼10× less rapidly than compounds with heterocycles or sulfide groups due to their modest electron donation capability. Heterocycles such as imidazole, furan, and thiazole also have increased conjugated-diene character with reduced aromaticity than phenols for facile [2 + 4] cycloadditions in the former. While ^1^O_2_ can also react with phenols via [2 + 4] cycloadditions across the 1,4 (*para*) positions, breaking of their greater aromatic content leads to reduced *k*_r_ values.^13^ For example, the *k*_r_ values for 4-acetylphenol is 1.5 × 10^6^ M^–1^ s^–1^ in H_2_O^59^ and for 4-acetyl-2,6-bis­(1,1-dimethylethyl)­phenol is 1.0 × 10^6^ M^–1^ s^–1^ in 1-butanol.^60^ Bisphenol A showed approximately one order of magnitude higher reaction rates with ^1^O_2_ compared to acetaminophen. This can be tentatively attributed to an electron-withdrawing carbonyl of the acetamide group dampening the ease for ^1^O_2_ addition in its phenyl ring compared to the electron-donating C­(Me_2_)­R group of bisphenol A. The sterics of the C­(Me_2_)­R group can dampen ^1^O_2_’s chemical reactivity in its own right. Notably, the ratio of our measured values of *k*_r_ between cimetidine and ranitidine (3.56) agrees well with the reported ratio value^37^ of 3.57.

Reaction rate constants in the literature can vary by more than an order of magnitude and are dependent on the pH of the solution. For example, the 4-acetylphenoxide anion in H_2_O (*k*_r_ = 2.4 × 10^7^ M^–1^ s^–1^) reacts 16 times faster than the corresponding acid form, 4-acetylphenol.^59^ In this context, we observe good agreement between our measured rate constants and those reported in the literature at different pH values as shown in Table 1. The overall reaction rate constants with APCs with ^1^O_2_ are in good agreement with previously reported studies,^37,42^ which are known to range from 10^4^ to 10^8^ M^–1^ s^–1^. Reaction rates for hydroxyl radicals (∼ 10^9^ M^–1^ s^–1^) are higher,^61^ yet suffer from diffusion distances of only a couple of bond lengths.

### 5.3 Bacteria Disinfection Study

Our results demonstrate that the photoreactor can inactivate bacteria with an inactivation efficiency of (2.15 × 10^–2^ %/J). Despite the high generation rate of singlet oxygen (3.13 mmol•h^–1^L^–1^), bacterial reduction achieved one log after 2 h of irradiation. The slow rate of inactivation may be due in part to diffusion limitations in the static photoreactor. Upon illumination, ^1^O_2_ is released from the surface but can diffuse <200 nm before being quenched to the ground state.^62^ Since the average distance between PMMA lightguides (i.e., fluid channel width) is up to 2 mm, only a small percentage of bacteria is sufficiently close to the PMMA surface to be inactivated initially, whereas the remainder of the bacteria must diffuse to the PMMA surface aided by limited convection induced by oxygen bubbling and/or thermal gradients. In a static solution, diffusion alone is too slow to contribute to inactivation of *E. coli* to an appreciable extent. The diffusion coefficient for an *E. coli* bacterium is estimated to be 5.13 × 10^–13^ m^2^ s^–1^ which would correspond to a diffusion length of 86 μm in 2 h. In contrast, UA and FFA reactions with ^1^O_2_ are not diffusion limited as these small molecules have diffusion coefficients that are 1.7 × 10^3^ times larger than *E. coli* and their diffusion lengths are estimated to be 3.1 × 10^3^ μm in 2 h (For calculations of diffusion coefficients and lengths, see Table S8).

Unlike UA or FFA, which requires only one successful collision with ^1^O_2_ to decompose the molecule, it is estimated that approximately 5 × 10^4 1^O_2_ molecules are required to inactivate a single bacterium (Table S9). This estimate is based on data from reference,^63^ which reports the number of ^1^O_2_ molecules needed to reduce mammalian fibrosarcoma cell survival to 1/e using BPD (benzoporphyrin derivative) as the photosensitizer is 4.6 × 10^7^. Therefore, we assume that bacteria inactivation in our static photoreactor system is limited by diffusion.

### 5.4 Scalability of the Photoreactor: Opportunities and Limitations

The proposed photoreactor concept is highly scalable due to its modular design and the materials of construction. The design enhances photon utilization and maximizes the illuminated surface area of the photosensitizer-coated regions. Because these lightguides are inherently leaky, the length of the lightguides is limited by scattering from the sidewall surfaces. Measurements of the normalized light scattered as a function of exposed roughened lightguide length (Figure S12) shows that the sidewall light emission decays exponentially with the length, with ∼ 10% of the total light remaining after a distance of 6 cm. By coupling LEDs to both ends of a roughened lightguide, the effective usable length can be extended to ∼ 12 cm, with a midpoint emission of ∼ 20% of the maximum sidewall emission. Furthermore, the amount of light emanating from the sidewalls is dependent upon the degree of surface roughness. If desired, a roughness gradient can be applied to the lightguide surface to generate a more uniform flux emanating along the length of the lightguide. Reactor capacity/throughput can be increased by incorporating a larger number of closely packed lightguides (e.g., a larger diameter reactor) and coupling LEDs to both ends of the lightguides to enable bidirectional light transmission. Coupling reactors in series would further increase system scale.

We anticipate that the reactor will be stable treating water for long periods of time. Preliminary work in our laboratory shows that the generation of singlet oxygen from the surface of polymer-supported PS surfaces prevents the adsorption of lipids, amino acids and proteins onto the surface. Moreover, we have shown that the ZnF_16_Pc PS is stable against photobleaching. The red LEDs are rated for ≥ 50,000 h of operation.

To achieve useful scale, the reactor may be modified to enable flow-through processing of water. Examples of flow-through systems have been described,^64^ where closely packed PMMA lightguides form narrow channels, reducing the diffusion distance of reactants and microorganisms to the ^1^O_2_ generating surfaces. Such narrow channels also enable alternative illumination schemes to be used.^64^ These designs are cost-effective because PMMA lightguides are inexpensive, inert, widely available and show excellent light-transmitting properties. The use of red light offers further advantages; red LEDs have been optimized for indoor agricultural applications with especially high energy efficiency, low cost and emission spectra that are well-aligned to absorption profiles of stable PSs.

## 6 Conclusion

This study presents an innovative approach to fabricating a PS-immobilized system for efficient ^1^O_2_ generation for photodegradation of APCs and disinfection. The PS, ZnF_16_Pc, was coated on the outer surface of PMMA lightguides by immersing the lightguides in an isopropyl alcohol solution of the PS. The immobilized PS is illuminated primarily by light coupled into the lightguide which is subsequently scattered along the sidewalls by surface roughening. A second source of illumination occurs through gaps between adjacent lightguides. The trapping rates of ^1^O_2_ were further increased by optimizing the loading of PS on the lightguide surface, and by demonstrating that excess PS loading does not increase ^1^O_2_ generation, which is important for reducing PS cost.

The effectiveness of the system was investigated by degrading a series of APCs as well as by bacterial disinfection studies. Destruction rates of APCs as fast as 4 × 10^–8^ mol/min were observed; however, the degradation rates were found to be dependent on the chemical structure of the compounds. Singlet oxygen reacts preferentially with compounds of lower aromaticity such as heterocycles compared to phenyl rings themselves, and also with compounds of higher nucleophilicity owing to ^1^O_2_’s electrophilicity.^65–68^ Bacteria can be inactivated in the photoreactor with the inactivation efficiency of 2.15 × 10^–2^ %/J. However, the bacterial reduction was limited to one log after 2 h, which is likely due to the slow diffusion of bacteria in the static photoreactor.

Our approach overcomes key limitations of existing ^1^O_2_ photoreactors, by generating large quantities of ^1^O_2_ (trapping rate of 3.1 mmol•h^–1^L^–1^ and steady-state concentration of 3.3 × 10^–11^ M) using a unique illumination system that is minimally affected by turbidity. This system is scalable and compatible with flow-through reactor designs with reduced material and operational costs. Future work will focus on increasing APC degradation rates, bacteria inactivation efficacy as well as the energy efficiency of flow-through reactors.

## Supplementary Material



## Notes

Quantification of PS coatings; PS leaching results; experimental setup for light measurements; LED emission and absorbance measurements; calculations of singlet oxygen trapping and steady-state concentrations; direct photolysis controls; contribution of ROS for APC degradation; light scattering measurements of smooth and roughened PMMA lightguides with and without PS coating; model for light delivery; UA and bacterial diffusion calculations; estimation of ^1^O_2_ collisions required to kill a bacterial cell; bacterial adsorption on PMMA lightguides; and light scattering measurements with the lightguide length, including the complete set of Figures S1–S12 and Tables S1–S9 (PDF)

## References

[1] Singh P. K., Kumar U., Kumar I., Dwivedi A., Singh P., Mishra S., Seth C. S., Sharma R. K. (2024). Critical Review
on Toxic Contaminants in Surface Water Ecosystem: Sources, Monitoring,
and Its Impact on Human Health. Environ. Sci.
Pollut. Res..

[2] Mishra R. K., Mentha S. S., Misra Y., Dwivedi N. (2023). Emerging Pollutants
of Severe Environmental Concern in Water and Wastewater: A Comprehensive
Review on Current Developments and Future Research. Water-Energy Nexus.

[3] Patel M., Kumar R., Kishor K., Mlsna T., Pittman C. U., Mohan D. (2019). Pharmaceuticals of Emerging Concern
in Aquatic Systems: Chemistry,
Occurrence, Effects, and Removal Methods. Chem.
Rev..

[4] Al-Abri M., Al-Ghafri B., Bora T., Dobretsov S., Dutta J., Castelletto S., Rosa L., Boretti A. (2019). Chlorination
Disadvantages and Alternative Routes for Biofouling Control in Reverse
Osmosis Desalination. Npj Clean Water.

[5] Summerfelt S. T. (2003). Ozonation
and UV Irradiation—an Introduction and Examples of Current
Applications. Aquac. Eng..

[6] Gonçalves A. A., Gagnon G. A. (2011). Ozone Application
in Recirculating Aquaculture System:
An Overview. Ozone Sci. Eng..

[7] Poyatos J. M., Muñio M. M., Almecija M. C., Torres J. C., Hontoria E., Osorio F. (2010). Advanced Oxidation
Processes for Wastewater Treatment:
State of the Art. Water. Air. Soil Pollut..

[8] Saravanan A., Deivayanai V. C., Kumar P. S., Rangasamy G., Hemavathy R. V., Harshana T., Gayathri N., Alagumalai K. (2022). A Detailed
Review on Advanced Oxidation Process in Treatment of Wastewater: Mechanism,
Challenges and Future Outlook. Chemosphere.

[9] Gullian M., Espinosa-Faller F. J., Núñez A., López-Barahona N. (2012). Effect of
Turbidity on the Ultraviolet Disinfection Performance in Recirculating
Aquaculture Systems with Low Water Exchange: Effect of Turbidity on
the Ultraviolet Disinfection Performance. Aquac.
Res..

[10] Carré E., Pérot J., Jauzein V., Lopez-Ferber M. (2018). Impact of
Suspended Particles on UV Disinfection of Activated-Sludge Effluent
with the Aim of Reclamation. J. Water Process
Eng..

[11] Mohaghegh
Montazeri M., Taghipour F. (2023). Operation of a High-Flow UV-LED Water
Treatment Reactor with Secondary Effluent for Stress Testing. Chem. Eng. J..

[12] Cheng G., Li Z., Sun L., Li Y., Fu J. (2020). Application of Microwave/Electrodeless
Discharge Ultraviolet/Ozone Sterilization Technology in Water Reclamation. Process Saf. Environ. Prot..

[13] Ghogare A. A., Greer A. (2016). Using Singlet Oxygen
to Synthesize Natural Products and Drugs. Chem.
Rev..

[14] Matsumoto, M. Synthesis with Singlet Oxygen. In Singlet O2; CRC Press: Boca Raton, FL, 1985; Vol. 2, pp 205–274.

[15] Zamadar, M. ; Greer, A. Singlet Oxygen as a Reagent in Organic Synthesis. In Handbook of Synthetic Photochemistry; Albini, A. , Fagnoni, M. , Eds.; Wiley, 2009; pp 353–386. 10.1002/9783527628193.ch11.

[16] Bartusik D., Aebisher D., Lyons A. M., Greer A. (2012). Bacterial
Inactivation
by a Singlet Oxygen Bubbler: Identifying Factors Controlling the Toxicity
of ^1^O_2_ Bubbles. Environ.
Sci. Technol..

[17] DellaGreca M., Iesce M. R., Previtera L., Purcaro R., Rubino M., Zarrelli A. (2008). Lignans by Photo-Oxidation of Propenyl Phenols. Photochem. Photobiol. Sci..

[18] Baptista M. S., Cadet J., Di Mascio P., Ghogare A. A., Greer A., Hamblin M. R., Lorente C., Nunez S. C., Ribeiro M. S., Thomas A. H., Vignoni M., Yoshimura T. M. (2017). Type I
and Type II Photosensitized Oxidation Reactions: Guidelines and Mechanistic
Pathways. Photochem. Photobiol..

[19] Durantini A. M., Greer A. (2021). Interparticle Delivery
and Detection of Volatile Singlet Oxygen at
Air/Solid Interfaces. Environ. Sci. Technol..

[20] DeRosa M. C., Crutchley R. J. (2002). Photosensitized Singlet Oxygen and Its Applications. Coord. Chem. Rev..

[21] Wang Y., Lin Y., He S., Wu S., Yang C. (2024). Singlet Oxygen: Properties,
Generation, Detection, and Environmental Applications. J. Hazard. Mater..

[22] Alves E., Faustino M. A. F., Neves M. G. P. M. S., Cunha Â., Nadais H., Almeida A. (2015). Potential Applications
of Porphyrins in Photodynamic
Inactivation beyond the Medical Scope. J. Photochem.
Photobiol. C.

[23] Nidheesh P. V., Boczkaj G., Ganiyu S. O., Oladipo A. A., Fedorov K., Xiao R., Dionysiou D. D. (2025). Generation, Properties, and Applications
of Singlet Oxygen for Wastewater Treatment: A Review. Environ. Chem. Lett..

[24] Kim H., Kim W., Mackeyev Y., Lee G.-S., Kim H.-J., Tachikawa T., Hong S., Lee S., Kim J., Wilson L. J., Majima T., Alvarez P. J. J., Choi W., Lee J. (2012). Selective
Oxidative Degradation of Organic Pollutants by Singlet Oxygen-Mediated
Photosensitization: Tin Porphyrin versus C_60_ Aminofullerene
Systems. Environ. Sci. Technol..

[25] Bartusik D., Aebisher D., Ghafari B., Lyons A. M., Greer A. (2012). Generating
Singlet Oxygen Bubbles: A New Mechanism for Gas–Liquid Oxidations
in Water. Langmuir.

[26] Manjón F., Santana-Magaña M., García-Fresnadillo D., Orellana G. (2014). Are Silicone-Supported
[C_60_]-Fullerenes
an Alternative to Ru­(II) Polypyridyls for Photodynamic Solar Water
Disinfection?. Photochem. Photobiol. Sci..

[27] Felgenträger A., Maisch T., Späth A., Schröder J. A., Bäumler W. (2014). Singlet Oxygen Generation in Porphyrin-Doped Polymeric
Surface Coating Enables Antimicrobial Effects on *Staphylococcus
Aureus*. Phys. Chem. Chem. Phys..

[28] García-Fresnadillo D. (2018). Singlet Oxygen
Photosensitizing Materials for Point-of-Use Water Disinfection with
Solar Reactors. ChemPhotoChem..

[29] Spagnul C., Turner L. C., Boyle R. W. (2015). Immobilized
Photosensitizers for
Antimicrobial Applications. J. Photochem. Photobiol.,
B.

[30] Hwang J.-W., Jung S.-J., Heo I., Son H.-A., Kim J.-H., Wang K.-K., Kim Y.-R. (2019). Study of
Singlet Oxygen Dynamics
on Silicon Polymer Matrix. J. Anal. Methods
Chem..

[31] Ihalagedara H. B., Xu Q., Greer A., Lyons A. M. (2025). High Singlet Oxygen Yields from a
Polymer-Supported Photosensitizer via Superhydrophobicity and Control
of Photosensitizer Morphology. Appl. Surf. Sci..

[32] Ihalagedara H. B., Xu Q., Greer A., Lyons A. M. (2024). Singlet Oxygen Generation on a Superhydrophobic
Surface: Effect of Photosensitizer Coating and Incident Wavelength
on ^1^O_2_ Yields. Photochem.
Photobiol..

[33] Satta G., Schirripa Spagnolo G., De Francesco E., Rampini L., Pieroni F., Leccese F. (2025). Red Light
in Underwater Measurement Applications: A
Short Review. Acta IMEKO.

[34] Bregnho̷j M., Dichmann L., McLoughlin C. K., Westberg M., Ogilby P. R. (2019). Uric Acid:
A Less-than-Perfect Probe for Singlet Oxygen. Photochem. Photobiol..

[35] Rabello B. R., Gerola A. P., Pellosi D. S., Tessaro A. L., Aparício J. L., Caetano W., Hioka N. (2012). Singlet Oxygen Dosimetry Using Uric
Acid as a Chemical Probe: Systematic Evaluation. J. Photochem. Photobiol. A.

[36] Appiani E., Ossola R., Latch D. E., Erickson P. R., McNeill K. (2017). Aqueous Singlet
Oxygen Reaction Kinetics of Furfuryl Alcohol: Effect of Temperature,
pH, and Salt Content. Environ. Sci. Process.
Impacts.

[37] Latch D. E., Stender B. L., Packer J. L., Arnold W. A., McNeill K. (2003). Photochemical
Fate of Pharmaceuticals in the Environment: Cimetidine and Ranitidine. Environ. Sci. Technol..

[38] Zuccato E., Calamari D., Natangelo M., Fanelli R. (2000). Presence of Therapeutic
Drugs in the Environment. Lancet.

[39] Kolpin D. W., Furlong E. T., Meyer M. T., Thurman E. M., Zaugg S. D., Barber L. B., Buxton H. T. (2002). Pharmaceuticals, Hormones, and Other
Organic Wastewater Contaminants in U.S. Streams, 1999–2000:
A National Reconnaissance. Environ. Sci. Technol..

[40] Fang W., Peng Y., Muir D., Lin J., Zhang X. (2019). A Critical
Review of Synthetic Chemicals in Surface Waters of the US, the EU
and China. Environ. Int..

[41] Aebisher D., Bartusik-Aebisher D., Belh S. J., Ghosh G., Durantini A. M., Liu Y., Xu Q., Lyons A. M., Greer A. (2020). Superhydrophobic Surfaces
as a Source of Airborne Singlet Oxygen through Free Space for Photodynamic
Therapy. ACS Appl. Bio Mater..

[42] Lee J., Von Gunten U., Kim J.-H. (2020). Persulfate-Based Advanced Oxidation:
Critical Assessment of Opportunities and Roadblocks. Environ. Sci. Technol..

[43] Li Y., Pan Y., Lian L., Yan S., Song W., Yang X. (2017). Photosensitized
Degradation of Acetaminophen in Natural Organic Matter Solutions:
The Role of Triplet States and Oxygen. Water
Res..

[44] Kreve S., Cândido Dos Reis A. (2025). Antibiofilm Capacity of PMMA Surfaces:
A Review of Current Knowledge. Microb. Pathog..

[45] Aebisher D., Zamadar M., Mahendran A., Ghosh G., McEntee C., Greer A. (2010). Fiber-optic Singlet
Oxygen [^1^O_2_ (^1^Δ_g_)] Generator Device Serving as a Point Selective
Sterilizer. Photochem. Photobiol..

[46] Aebisher D., Bartusik D., Liu Y., Zhao Y., Barahman M., Xu Q., Lyons A. M., Greer A. (2013). Superhydrophobic Photosensitizers.
Mechanistic Studies of ^1^O_2_ Generation in the
Plastron and Solid/Liquid Droplet Interface. J. Am. Chem. Soc..

[47] Peterson B. M., McNally A. M., Cory R. M., Thoemke J. D., Cotner J. B., McNeill K. (2012). Spatial and Temporal
Distribution of Singlet Oxygen
in Lake Superior. Environ. Sci. Technol..

[48] Latch D. E., McNeill K. (2006). Microheterogeneity
of Singlet Oxygen Distributions
in Irradiated Humic Acid Solutions. Science.

[49] Grandbois M., Latch D. E., McNeill K. (2008). Microheterogeneous Concentrations
of Singlet Oxygen in Natural Organic Matter Isolate Solutions. Environ. Sci. Technol..

[50] Cai L., Yao Q., Du X., Zhong J., Lu H., Tao X., Zhou J., Dang Z., Lu G. (2023). Validation of Quenching
Effectiveness and Pollutant Degradation Ability of Singlet Oxygen
through Model Reaction System. J. Hazard. Mater..

[51] Baptista M. S., Cadet J., Greer A., Thomas A. H. (2023). Practical Aspects
in the Study of Biological Photosensitization Including Reaction Mechanisms
and Product Analyses: A Do’s and Don’ts Guide. Photochem. Photobiol..

[52] Griesbeck A. G., de Kiff A., Kleczka M. (2014). Tetraphenylporphyrin-Catalyzed
Tandem
Photooxygenation of Polyenes and 1,4-Dienes: Multiple and Diverse
Oxyfunctionalizations. Adv. Synth. Catal..

[53] Sofikiti N., Tofi M., Montagnon T., Vassilikogiannakis G., Stratakis M. (2005). Synthesis of the Spirocyclic Core of the Prunolides
Using a Singlet Oxygen-Mediated Cascade Sequence. Org. Lett..

[54] Nahar K., Essang S., Lapoot L., Greer A. (2025). Tandem Singlet Oxygenation:
Regioselective Reaction of Two ^1^O_2_ Molecules
by a Nonconjugated Diprenyl Phenol. Photochem.
Photobiol..

[55] Egorov S. Y., Kurella E. G., Boldyrev A. A., Krasnovsky A. A. (1992). The Quenching of Singlet Molecular-Oxygen
by Carnosine
and Anserine in Aqueous-Solution. Bioorg. Khim..

[56] Michaeli A., Feitelson J. (1994). Reactivity
of Singlet Oxygen Toward Amino Acids and
Peptides. Photochem. Photobiol..

[57] Wilkinson F., Helman W. P., Ross A. B. (1995). Rate Constants
for the Decay and
Reactions of the Lowest Electronically Excited Singlet State of Molecular
Oxygen in Solution. An Expanded and Revised Compilation. J. Phys. Chem. Ref. Data.

[58] Clennan E. L., Greer A. (1996). Effect of Alcohols
on the Photooxidative Behavior of Diethyl Sulfide. J. Org. Chem..

[59] Tratnyek P. G., Hoigne J. (1991). Oxidation of Substituted
Phenols in the Environment:
A QSAR Analysis of Rate Constants for Reaction with Singlet Oxygen. Environ. Sci. Technol..

[60] Snyakin A. P., Samsonova L. V., Shlyapintokh V. Ya., Ershov V. V. (1978). Kinetics and Mechanism
of the Interaction of Sterically Hindered Phenols with Singlet Oxygen. Bull. Acad. Sci. USSR Div. Chem. Sci..

[61] Yan Y., Wei Z., Duan X., Long M., Spinney R., Dionysiou D. D., Xiao R., Alvarez P. J. J. (2023). Merits and Limitations of Radical
vs. Nonradical Pathways in Persulfate-Based Advanced Oxidation Processes. Environ. Sci. Technol..

[62] Zamadar M., Aebisher D., Greer A. (2009). Singlet Oxygen
Delivery Through the
Porous Cap of a Hollow-Core Fiber Optic Device. J. Phys. Chem. B.

[63] Zhu T. C., Kim M. M., Liang X., Finlay J. C., Busch T. M. (2015). In-Vivo
Singlet Oxygen Threshold Doses for PDT. Photonics
Lasers Med..

[64] Mahmudov, R. ; Ihalagedara, H. B. ; Lyons, A. M. ; Greer, A. ; Xu, Q. System and Method of Generating Singlet Oxygen for Water Purification. PCT/US2025/015890, 2025.

[65] Iyer A., Lapoot L., Greer A. (2025). Mechanistic
and Curtin–Hammett
Studies of the ^1^O_2_ Oxidation of a Prenyl Phenol
and Phenolate Anion. J. Phys. Org. Chem..

[66] Kernan M.
R., Faulkner D. J. (1988). Regioselective
Oxidation of 3-Alkylfurans to 3-Alkyl-4-Hydroxybutenolides. J. Org. Chem..

[67] Murray R. W., Agarwal S. K. (1984). Singlet Oxygen Oxidation
of Substituted Thiobenzamides. J. Photochem..

[68] Brecht R., Büttner F., Böhm M., Seitz G., Frenzen G., Pilz A., Massa W. (2001). Photooxygenation
of the Helimers
of (−)-Isocolchicine: Regio- and Facial Selectivity of the
[4 + 2] Cycloaddition with Singlet Oxygen and Surprising Endoperoxide
Transformations. J. Org. Chem..

