# Supplementary material for: A Scalable Singlet Oxygen Reactor for Photodegradation of Active Pharmaceutical Compounds and Disinfection
Source: ACS ES T Water. 2026 Mar 6;6(4):2068–79. doi: 10.1021/acsestwater.5c00915 (PMC13077686; doi:10.1021/acsestwater.5c00915)
Supplement: Supplementary file 1 [file ew5c00915_si_001.pdf]

A Scalable Singlet Oxygen Reactor for Photodegradation of Active Pharmaceutical Compounds and Disinfection

Pabasara Samarawickrama[1,2†], Hasanuwan Ihalagedara[1,2†], QianFeng Xu[3], Christine Boisrobert[4], Rovshan Mahmudov[4*], Alexander Greer[2,3,5*], Alan Lyons[1,2,3*]

1. Department of Chemistry, College of Staten Island, City University of New York, Staten Island, New York 10314, United States
2. Ph.D. Program in Chemistry, The Graduate Center of the City University of New York, New York, New York 10016, United States
3. SingletO2 Therapeutics LLC, 211 Warren St., Newark, New Jersy 07103, United States
4. Air Liquide Innovation Campus Delaware, 200 Gbc Dr., Newark, Delaware 19702, United States
5. Department of Chemistry, Brooklyn College, City University of New York, Brooklyn, New York 11210, United States

† Pabasara Samarawickrama and Hasanuwan Ihalagedara contributed equally to this work.

* Email: rovshan.mahmudov@airliquide.com

* Email: agreer@brooklyn.cuny.edu

* Email: alan.lyons@csi.cuny.edu

Table of Content

List of Sections

List of Sections	Page
1. Assessing the coating concentration on roughened lightguides	S5
2. Photosensitizer leaching experiment	S5
3. Light measurements	S5
4. Absorbance of $ZnF_{16}Pc$ and the emission of LED light sources	S5
5. Estimating the amount of $^1O_2$ trapped in the reactor	S6
6. Steady state concentration of $^1O_2$ calculations	S6
7. Direct photolysis control experiments for APCs	S6
8. Contribution of ROS for APC degradation	S6
9. Effect of surface roughness on incident light scattering	S6
10. Effect of PS coating on light scattering	S7
11. The model for light delivery	S7
12. Estimating diffusion distance of UA molecule vs a bacterium in water	S8
13. Estimating the number of $^1O_2$ collisions required to kill a bacteria cell	S8
14. Bacteria adsorption on roughened PMMA lightguides	S8
15. Light scattering measurements as a function of the lightguide length	S9

Table of Content

List of Figures	Page
**Figure S1.** Calibration curve obtained for different concentration of $ZnF_{16}Pc$/acetone solutions	S10
**Figure S2.** Uric acid 291 nm peak before and after 10 minutes of irradiation ($\geq$ 5 trials). The absorbance value at 291 nm peak was 1.02 before the irradiation and 1.01 after irradiation with standard deviation of $\leq \pm 0.01$	S10
**Figure S3**. Schematics of a) integrating sphere setup to measure light intensity of lightguides b) scattering of light from smooth vs roughened lightguides	S11
**Figure S4.** Absorption spectra of $ZnF_{16}Pc$ (blue-dotted trace) and emission spectra of green LED (green trace) and red LED (red trace)	S11
**Figure S5.** First order plot of FFA degradation as a function of irradiation time. The total irradiation time was 300 s with data acquired every 60 s (n = 6 time points). Error bars represent standard deviation values calculated from three independent trials	S12
**Figure S6.** Direct photolysis control experiments performed under 637 nm LED irradiation (300 mW cm^{-2}) for ranitidine, cimetidine, and famotidine using uncoated roughened lightguides	S12
**Figure S7.** First order plots of a) 100 µM of pure cimetidine and b) 100 µM of pure FFA with D-mannitol and sodium azide (NaN_3)	S13
**Figure S8.** Red LED emission spectra coupled to a) smooth lightguides at 2000 ms integrating time b) smooth lightguides at 1000 ms integrating time c) roughened lightguides at 2000 ms integrating time and d) roughened lightguides at 1000 ms integrating time with and without the end cap and with and without the PS	S14
**Figure S9**. Green LED emission spectra when coupled to a) smooth lightguides and b) roughened lightguides with and without the end cap and with and without the PS at 2000 ms integrating time	S15
**Figure S10.** Schematic showing the % light transmission through the PMMA lightguides (smooth-left and roughened-right) and in between the lightguides	S15
**Figure S11.** Control experiments measuring the precent reduction of bacteria as a function of light dose/time using roughened PMMA lightguides without PS: lightguides with no PS but with red light, PS- L+ (green points); and lightguides without PS and without red light, PS- L- (purple points)	S16
**Figure S12.** a) Schematic illustration of a 12 cm long lightguide coupled to red LED and fully inserted into an integrating sphere, where the exposed emitting length (2-12 cm) is controlled by masking b) Normalized sidewall emission intensity at λ_{max}= 639 nm from a roughened lightguide as a function of exposed length	S16

Table of Content

List of Tables Page

Table S1. Stepwise calculations to determine PS surface coating on a single lightguide S17

Table S2. Example calculation on the trapping rate of $^1O_2$ using uric acid S17

Table S3. The rates of cimetidine and FFA degradation with and without the presence of inhibitors S17

Table S4. Stepwise calculation of the steady-state concentration of singlet oxygen ($^1O_2$) based on the observed rate constant (k_{obs}) and the reaction rate constant (k_r) S18

Table S5. Effect of PS coating on red light transmission through lightguide surfaces. Intensity at 637 nm from the red LED transmitted through the lightguides including emission from all surfaces including the sidewalls and end (No cap) as well as only the sidewalls (Capped) for both smooth and roughened lightguides with and without PS. Intensity measurements were taken by at least three different freshly coated/uncoated lightguides. Measuring at two different integration times (1000 and 2000 ms) demonstrates the reproducibility of the measurements S18

Table S6. Effect of PS coating on green light transmission through lightguide surfaces. Intensity at 509 nm from the green LED transmitted through the lightguides including emission from all surfaces including the sidewalls and end (No cap) as well as only the sidewalls (Capped) for both smooth and roughened lightguides with and without PS. Intensity measurements were taken by at least three different freshly coated/uncoated lightguides S19

Table S7. Comparison of the intensities of the maximum wavelengths of red and green LED emission coupled to the PS coated lightguides with no cap. The standard deviation represents at least three different measurements S19

Table S8: Diffusion coefficient calculation of UA molecule vs *E.coli* bacterium in water S20

Table S9. Estimating the number of $^1O_2$ collisions required to kill *E. coli* bacterium S20

1. Assessing the coating concentration on roughened lightguides

The amount of $ZnF_{16}Pc$ immobilized on a single lightguide was quantified by extracting the coated PS into acetone and measuring the absorbance at 637 nm, as described in Section 3.1. The step-by-step calculations used to estimate the surface loading are summarized in Table S1 using the calibration curve shown in Figure S1.

2. Photosensitizer leaching experiment

To evaluate potential leaching of PS from the lightguide surfaces, an experiment was carried out by immersing 50 freshly prepared PS-coated lightguides in 60 mL of PBS buffered solution and soaked overnight. The extract was analyzed using UV-vis spectroscopy to detect the presence of PS in the leachate. No PS was detected by monitoring the maximum absorbance peak at 639 nm. To further confirm that no PS was leached, the same PBS buffer was used to prepare an 800 μM uric acid (UA) solution. To assess $^1O_2$ from the leachate, 15 pristine (PS-free) lightguides were immersed in 2 mL of the prepared UA containing leachate solution. The maximum absorbance peak for UA (291 nm) was monitored after 10 minutes of irradiation by extracting 100 μL of the irradiated UA solution and diluting it with 900 μL of PBS (irradiation was done using a red LED with 100 mW/cm^2 incident irradiance). The experiment was repeated over 5 trials and no evidence of $^1O_2$ generation was observed as shown in Figure S2.

3. Light measurements

The delivery of light through the lightguides was quantified using a 6" integrating sphere, with lightguides coupled to a red or green LED at one end and the other end fully inserted into the integrating sphere, as shown in Figure S3a. The intensity of light exiting from the lightguide was monitored with and without an end cap to quantify the sidewall scattering versus straight-through delivery for both smooth and roughened lightguides as schematically shown in Figure S3b.

4. Absorbance of $ZnF_{16}Pc$ and the emission of LED light sources

The absorbance of $ZnF_{16}Pc$ and the emission of LED light sources are shown in Figure S4. The red LED (λ_{max}= 637 nm) has the maximum overlap with the PS absorbance while green LED (λ_{max}= 509 nm) shows minimal overlap.

5. Estimating the amount of $^1O_2$ trapped in the reactor

The amount of $^1O_2$ trapped by the 800 μM UA solution in the photoreactor was calculated by the stepwise calculations summarized in Table S2.

6. Steady state concentration of $^1O_2$ calculations

The steady state concentration $[^1O_2]_{ss}$ was calculated by using first order decay kinetics of FFA as a reference based on the equation[1] $k_{obs} / k_r = [^1O_2]_{ss}$. The k_{obs} was obtained from the slope of the linear plot of ln[FFA] vs time shown in Figure S5. The calculation of $[^1O_2]_{ss}$ for roughened lightguides used for APC degradation is shown in Table S4.

7. Direct photolysis control experiments for APC

The direct photolysis control experiments were performed for the APCs famotidine, ranitidine and cimetidine using a red LED under the same experimental conditions as described in experimental section 3.5, but without the PS coating. As shown in the Figure S6, the degradation of these compounds was minimal (1–2%) under light irradiation without PS after 480 s of irradiation, confirming that direct photolysis plays a negligible role in the overall degradation process.

8. Contribution of ROS for APC degradation

To assess the formation of ROS, commonly standard quenchers such as sodium azide (NaN_3, 2 mM) and D-mannitol (200 μM) were used in pure cimetidine and FFA (100 μM each) in aqueous phosphate buffered saline, as shown in Figure S7 and Table S3. Sodium azide caused 61% and 43% reduction of the rates of cimetidine and FFA, respectively while D-mannitol showed no significant reduction of rates in both compounds. Quantitative analyses for both the pure compounds and their equimolar mixture (FFA: cimetidine, 1:1) are summarized in Table S3.

9. Effect of surface roughness on incident light scattering

The light transmission through the lightguides was first studied with the red LED. To quantify sidewall scattering, the lightguides were capped on one end to block straight-through light delivery, allowing light to scatter through the sidewall. The results showed a 94% drop in light intensity for smooth lightguides (Figure S8a) upon capping, while only a 5% drop was observed

for roughened lightguides (Figure S8c). However, when the same measurements were carried out with PS-coated lightguides, the light intensities were dropped by 29% and 25% for smooth and roughened lightguides (Figure S8a and S8c) respectively, indicating that PS particles on the sidewalls absorb red light regardless of the sidewall texture.

To validate the accuracy of the measurements, the intensities were repeated with the different integrating time (1000 ms, Figure S8b,d) and the similar results (% intensity reduction) were obtained as shown in Table S5.

10. Effect of PS coating on light scattering

To study the effect of light scattering from the sidewall bound PS, the light intensity measurements were taken using a green LED which has minimal absorbance by $ZnF_{16}Pc$. Upon capping, both the smooth lightguides (with and without PS) (Figure S9a) showed similar % reduction (90%). Roughened lightguides (Figure S9b) also showed similar reduction with 6.1% reduction showing no significant scattering by PS. A quantitative analysis of green light transmission is shown in Table S6.

A summary of the light intensity difference between lightguides with PS is shown in Table S7. The light intensity was dropped by 25% and 29% when PS was coated on both smooth and roughened lightguides respectively. The % drop was minimal when green light was coupled indicating that the light scattering from PS particles on sidewalls is minimal.

11. The model for light delivery

Not all the incident light transmitted through the lightguides contributes solely to $^1O_2$ generation. To quantify the percentage of light transmittance through the lightguides, a controlled experiment was conducted in which the roughened lightguides' ends were either polished (to facilitate light transmittance) or darkened (to block light), and the UA degradation was measured under both conditions. When the polished ends were darkened, UA degradation was reduced by 57%, indicating that 43% of the incident light is transmitted between the lightguides. The model for light delivery for the whole system (smooth and roughened lightguides) is shown in Figure S10.

12. Estimating diffusion distance of UA molecule vs a bacterium in water

The diffusion coefficient and the average diffusion length travelled (after 2 hrs.) by a UA molecule and a *E.coli* bacterium was calculated based on the following equations as summarized in Table S8.

Diffusion Coefficient: $D = \frac{K\beta\,T}{6\pi\eta r}$

Root Mean Square Displacement: $\sqrt{(X^2)} = \sqrt{2Dt}$

13. Estimating the number of $^1O_2$ collisions required to kill a bacteria cell

Reference[2] estimates that approximately 4.6×10^7 singlet oxygen ($^1O_2$) molecules per cell are needed to reduce mammalian cell survival to 1/e when using BPD as the photosensitizer in a laser-induced ROS mouse model. Mammalian fibrosarcoma cells typically range from 10–20 µm in diameter. For this estimation, we assume a diameter of 10 µm. In comparison, an *E. coli* bacterium is about 1 µm in diameter. Assuming both cell types are spherical, the volume of a mammalian cancer cell is roughly 1,000 times greater than that of an *E. coli* cell. Scaling the singlet oxygen requirement accordingly, we estimate that approximately 5×10^4 $^1O_2$ molecules per bacterial cell are needed to reduce survival to 1/e.

14. Bacteria adsorption on roughened PMMA lightguides

A set of control experiments were carried out using lightguides that were not coated with the photosensitizer. Two systems were studied, one using red LED illumination, PS- L+, (using the same illumination conditions used for the bacteria inactivation study shown in Figure 7) and one without LED light, PS- L-. For clarity, only the PS- L+ data are plotted in Figure 7. Figure S11 shows both data sets plotted in the same graph. The results demonstrate that the reduction in CFU levels is similar for cases under red LED illumination as well as those without illumination. The reduction in CFU levels of E. coli are consistent with the absorption of bacteria on roughened PMMA surfaces.

The experiments were performed with no PS on surface-roughened PMMA lightguides with bacterial suspensions of 1×10^5 CFU/mL. The bacteria suspensions were maintained at temperatures between 24-28°C and oxygen (~90%) was continuously bubbled throughout the experiment. Samples (50 μL) were extracted at different time intervals (30, 60, 90, 120 min). Serial dilutions in sterile PBS were plated on Luria agar (100 μL) and incubated at 37°C for ~15 h, followed by colony counting.

15. Light scattering measurements as a function of the lightguide length

To measure the sidewall light scattering as a function of lightguide length, a 12 cm long roughened lightguide, capped at the end, was fully inserted into the integrating sphere and the red LED (λ_{max}= 639 nm) was coupled to the external end. The sidewall emission at the maximum intensity of 639 nm was quantified as a function of exposed length by masking (taping) the lightguide to restrict the emission to defined lengths (2-12 cm) as shown in Figure S12a.

Based on the normalized light intensity measurements (Figure S12b), the sidewall emission increases rapidly near the light input and approaches a plateau with increasing distance along the lightguide. Approximately 46% of total sidewall emission is extracted within the first 2 cm, and ~72% within the first 4 cm while increasing the exposed length to 6 cm results in ~90% of the total emission. Revealing the additional 6 cm length of the lightguide results in a meager 8% additional light. Coupling LEDs to both ends of a lightguide would allow this length to be doubled to ~12 cm, with the midpoint now receiving ~20% of the maximum sidewall emission. We believe that this 12 cm length can be effectively used in water treatment applications. See the full manuscript for additional details regarding scaling strategies.

Figures

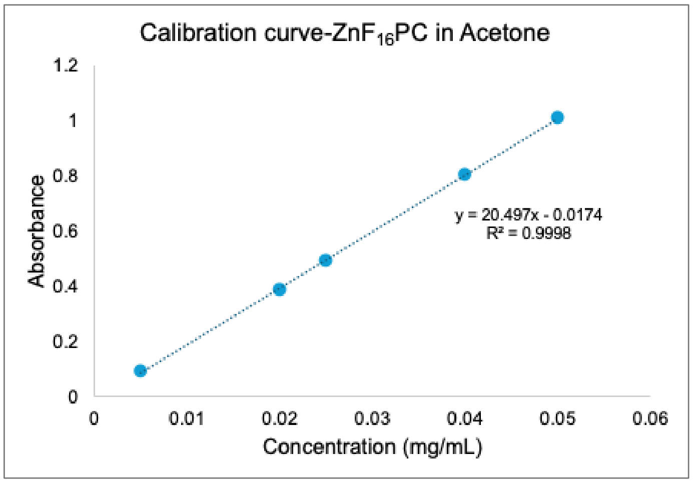

Figure S1. Calibration curve obtained for different concentration of $ZnF_{16}Pc$/acetone solutions

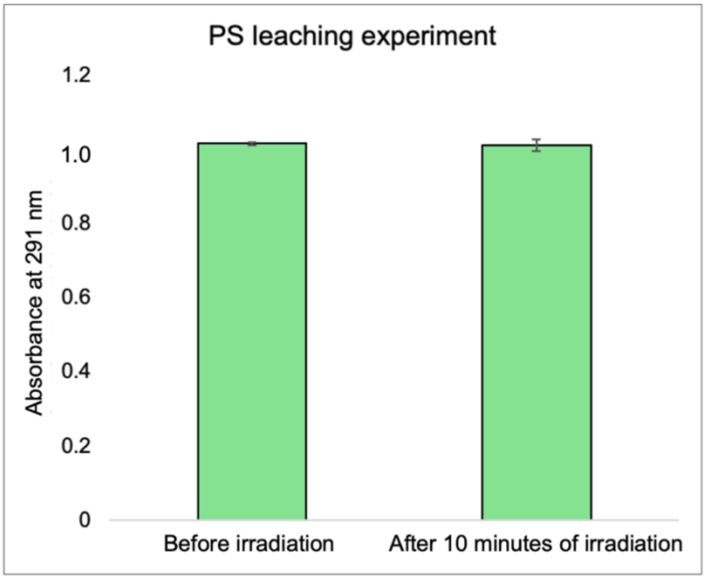

Figure S2. Uric acid 291 nm peak before and after 10 minutes of irradiation (≥ 5 trials). The absorbance value at 291 nm peak was 1.02 before the irradiation and 1.01 after irradiation with standard deviation of $\leq \pm 0.01$

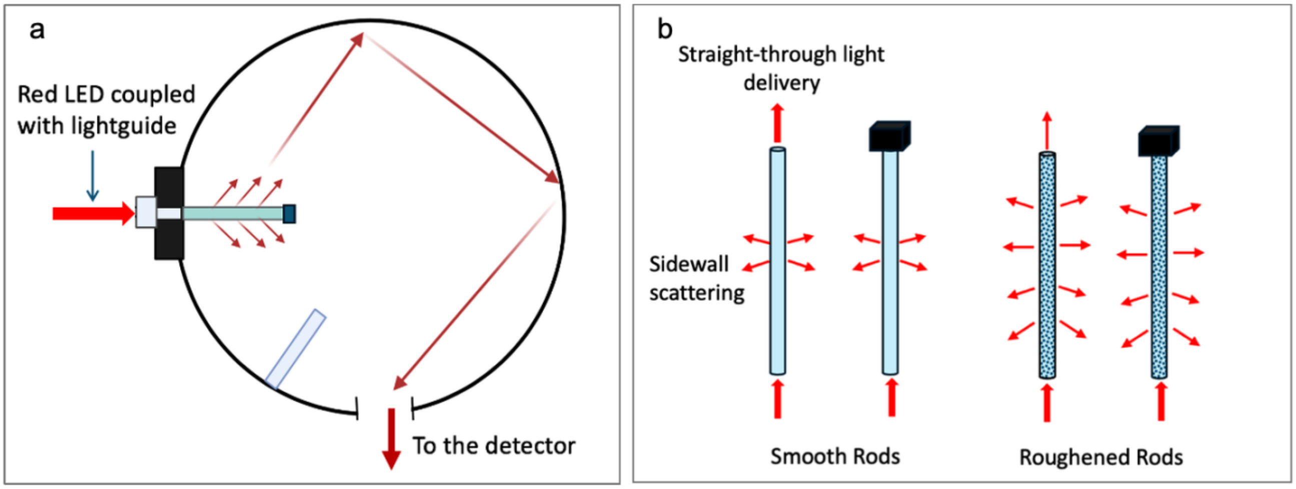

Figure S3. Schematics of a) integrating sphere setup to measure light intensity of lightguides b) scattering of light from smooth vs roughened lightguides

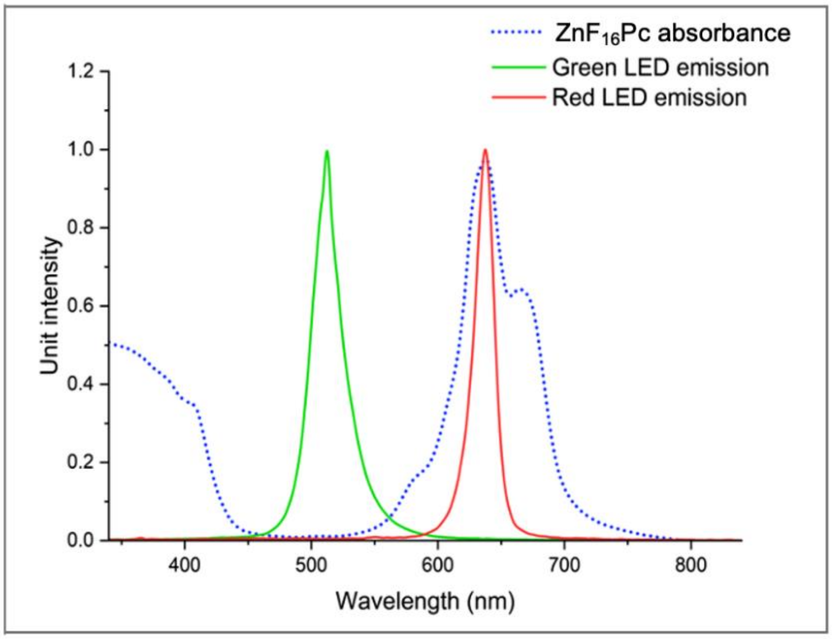

Figure S4. Absorption spectra of $ZnF_{16}Pc$ (blue-dotted trace) and emission spectra of green LED (green trace) and red LED (red trace)

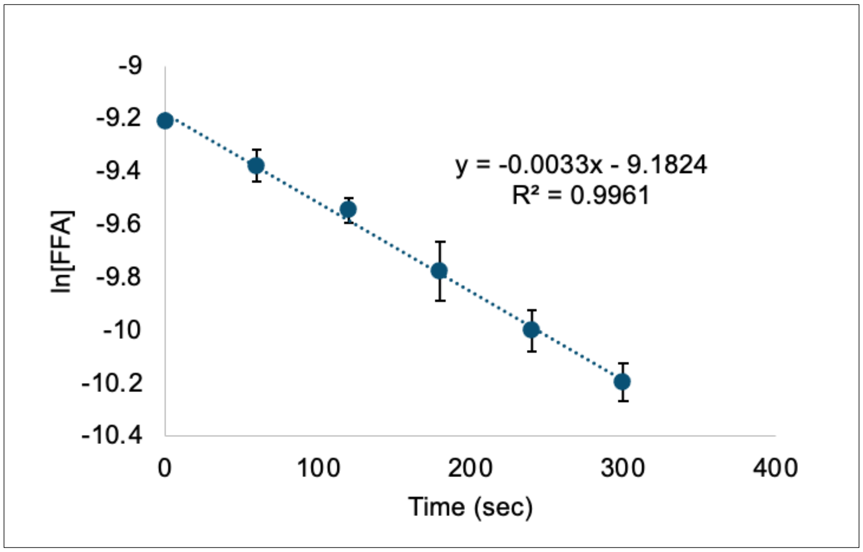

Figure S5. First order plot of FFA degradation as a function of irradiation time. The total irradiation time was 300 s with data acquired every 60 s (n = 6 time points). Error bars represent standard deviation values calculated from three independent trials

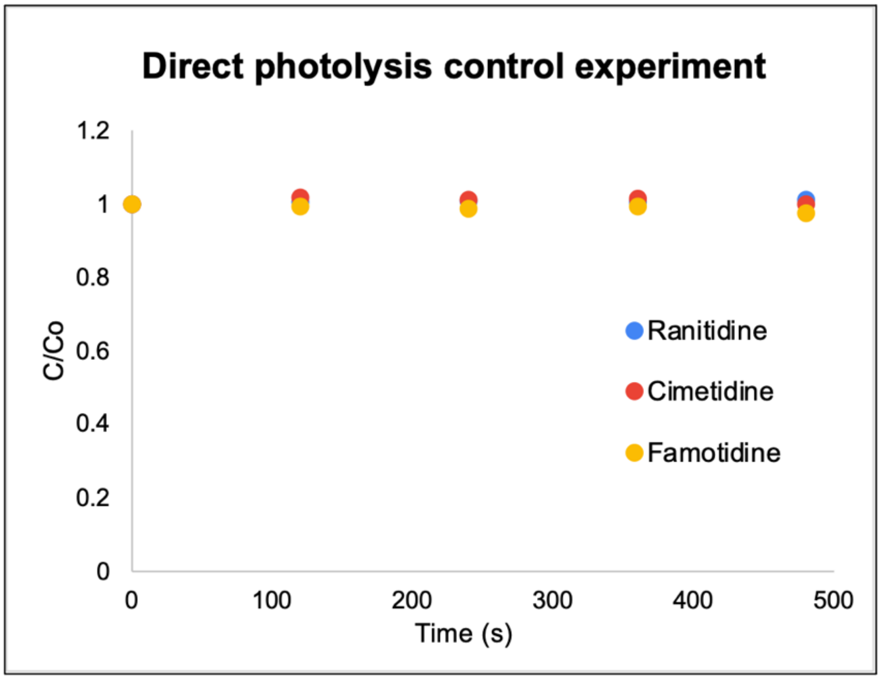

Figure S6. Direct photolysis control experiments performed under 637 nm LED irradiation (300 mW cm^{-2}) for ranitidine, cimetidine, and famotidine using uncoated roughened lightguides

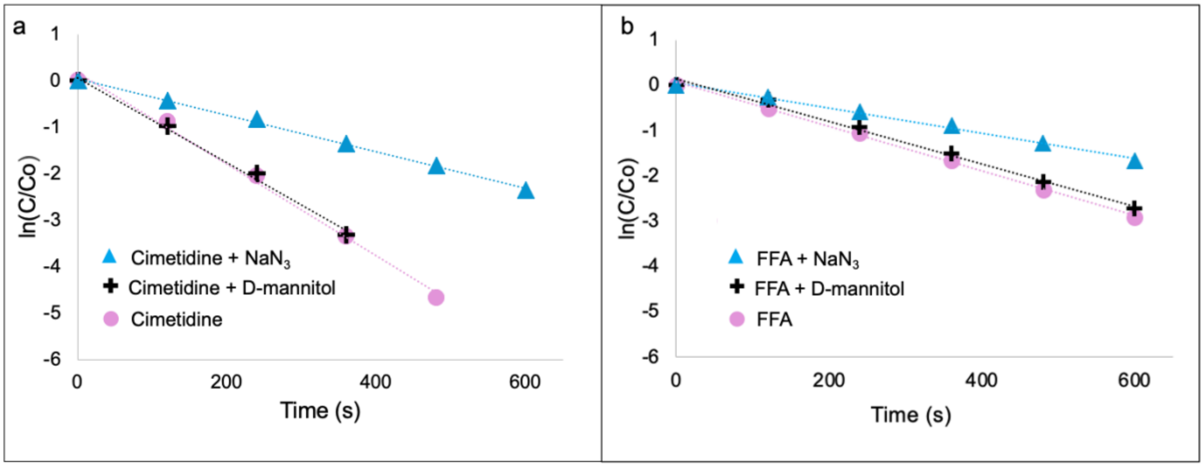

Figure S7. First order plots of a) 100 µM of pure cimetidine and b) 100 µM of pure FFA with D-mannitol and sodium azide (NaN_3). Each plot exhibited an R^2 value >0.99

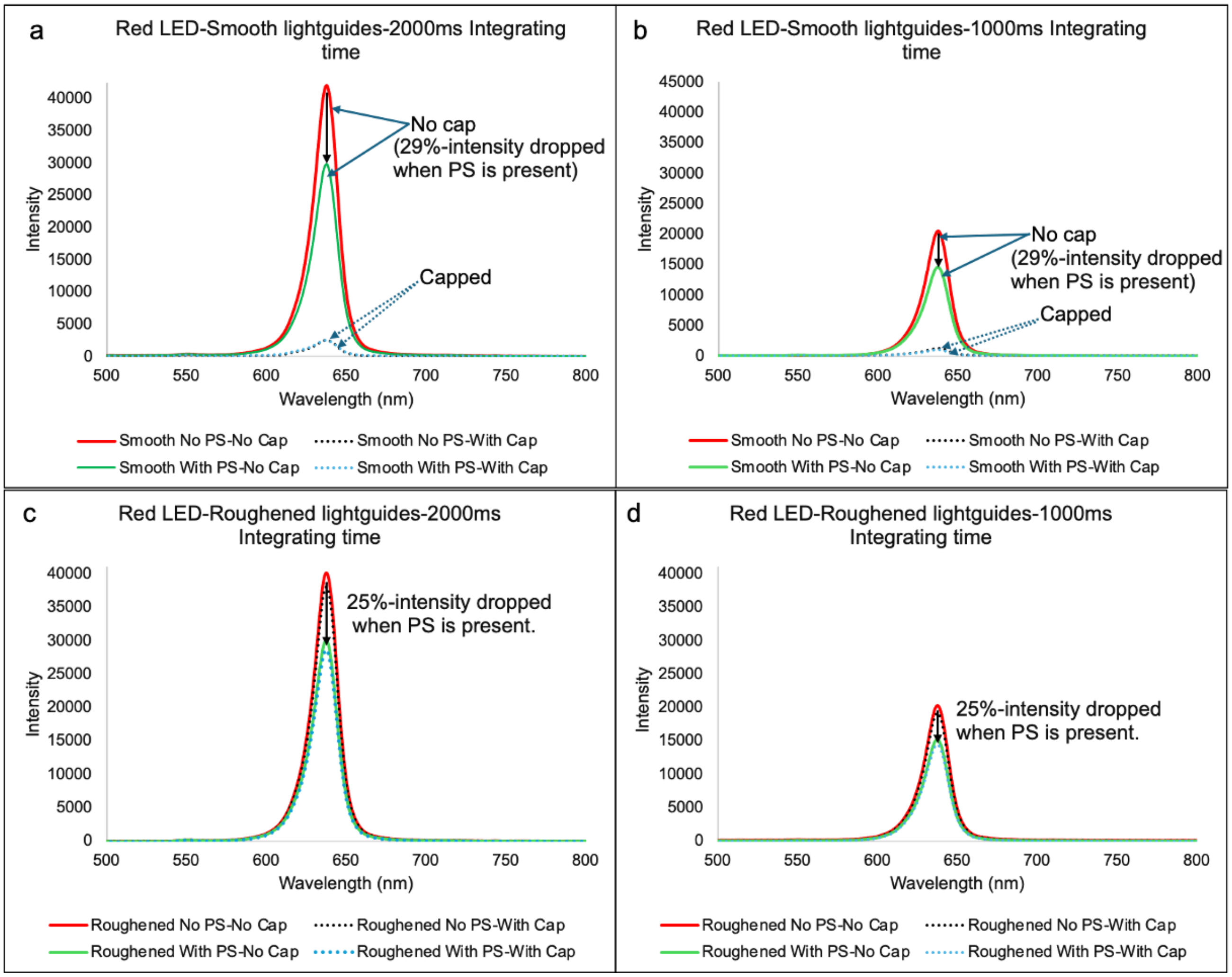

Figure S8. Red LED emission spectra coupled to a) smooth lightguides at 2000 ms integrating time b) smooth lightguides at 1000 ms integrating time c) roughened lightguides at 2000 ms integrating time and d) roughened lightguides at 1000 ms integrating time with and without the end cap and with and without the PS

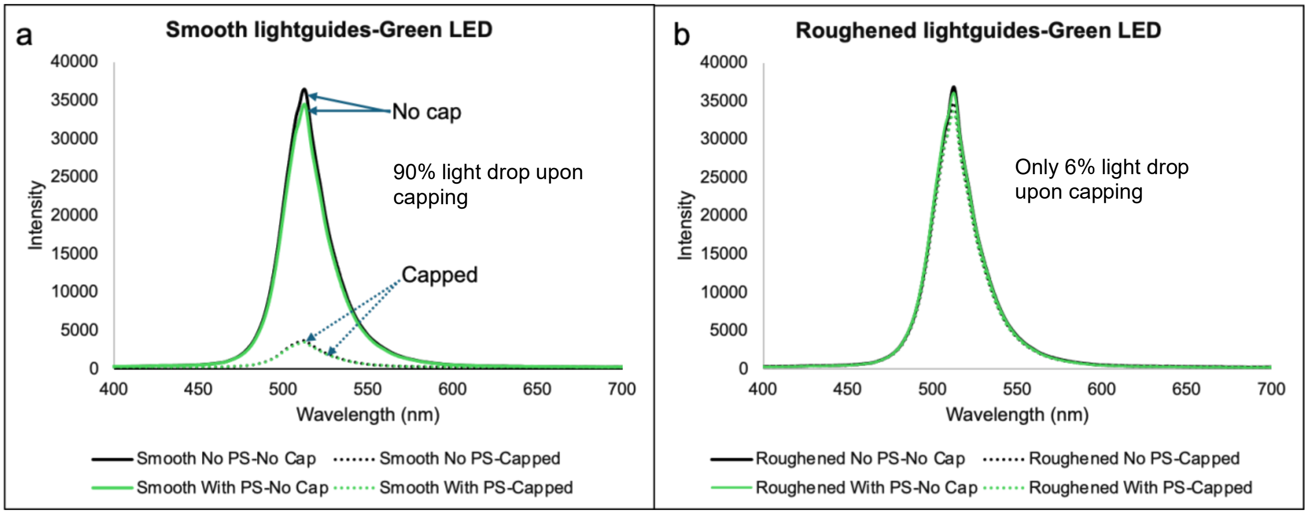

Figure S9. Green LED emission spectra when coupled to a) smooth lightguides and b) roughened lightguides with and without the end cap and with and without the PS at 2000 ms integrating time

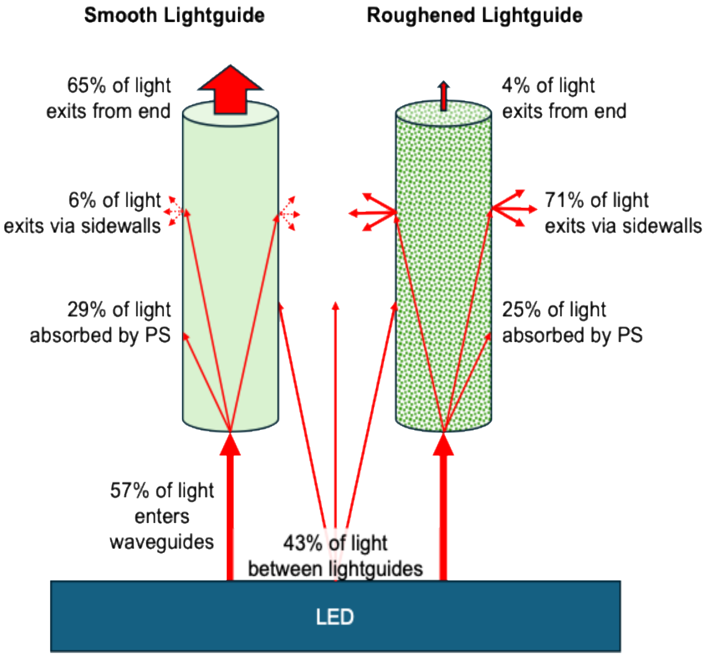

Figure S10. Schematic showing the % light transmission through the PMMA lightguides (smooth-left and roughened-right) and in between the lightguides

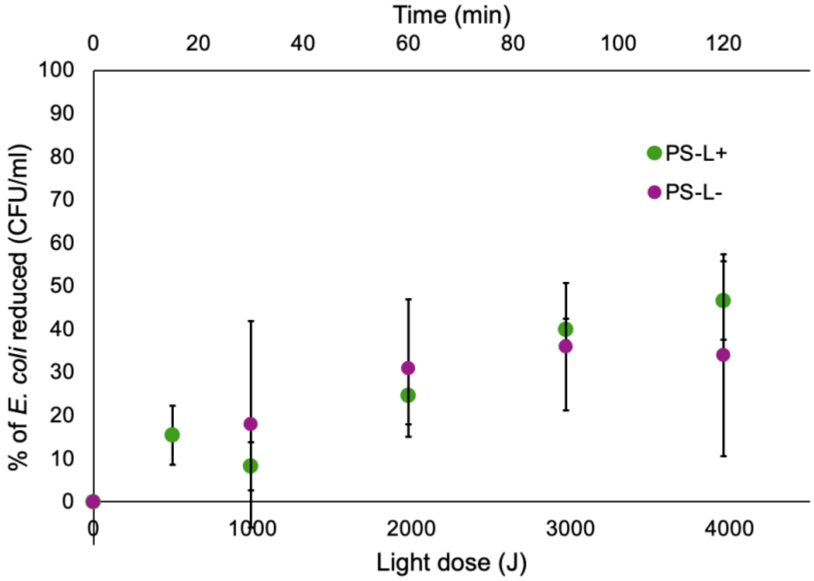

Figure S11. Control experiments measuring the precent reduction of bacteria as a function of light dose/time using roughened PMMA lightguides without PS: lightguides with no PS but with red light, PS- L+ (green points); and lightguides without PS and without red light, PS- L- (purple points)

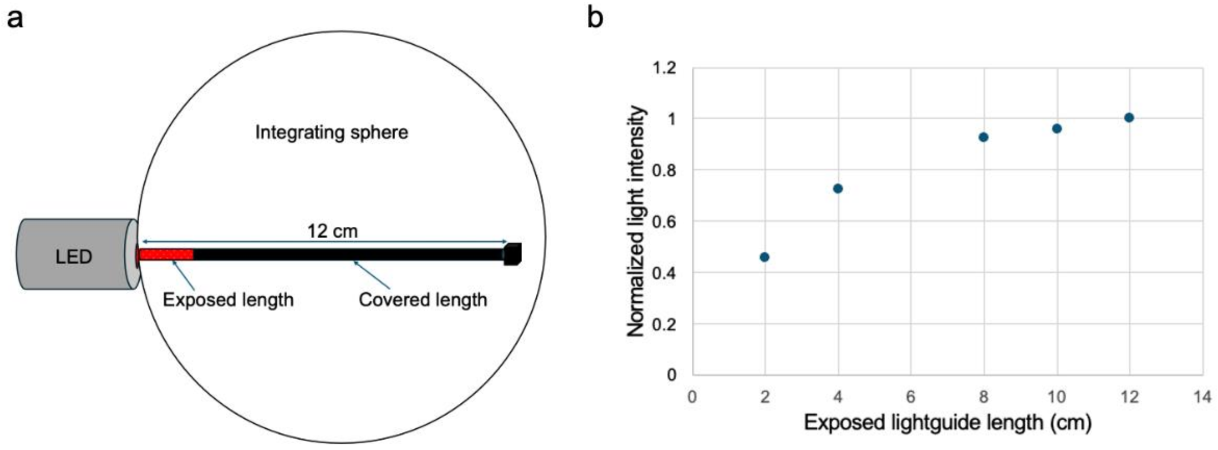

Figure S12. a) Schematic illustration of a 12 cm long lightguide coupled to red LED and fully inserted into an integrating sphere, where the exposed emitting length (2-12 cm) is controlled by masking b) Normalized sidewall emission intensity at λ_{max}= 639 nm from a roughened lightguide as a function of exposed length

Tables

Table S1. Stepwise calculations to determine PS surface coating on a single lightguide

Parameter	Value
Absorbance value	0.26
Concentration of $ZnF_{16}Pc$/IPA from calibration curve	0.013 mg/mL
Mass of $ZnF_{16}Pc$ in 5 mL of Acetone	0.066 mg
Mass of $ZnF_{16}Pc$ on a single lightguide	0.0022 mg
Surface area of a single lightguide	3.1 cm^2
Mass of $ZnF_{16}Pc$ per area	7.1×10^{-4} mg/cm^2

Table S2. Example calculation on the trapping rate of $^1O_2$ using uric acid

Parameter	Value
UA concentration	8.03×10^{-4} mol/L
Volume of test solution in the reactor	2.00 mL
Total amount of UA in the reactor before irradiation	1.61×10^{-6} mol
Percentage of UA reacted in 10 minutes	65%
Calculated amount of $^1O_2$ trapped in 10 min in 2 ml of solution	1.04×10^{-6} mol
Calculated amount of $^1O_2$ trapped per hour per liter	3.13 mmol•$h^{-1}L^{-1}$

Table S3. The rates of cimetidine and FFA degradation with and without the presence of inhibitors

	Without inhibitors (s^{-1}) x10^{-2}	D-mannitol (s^{-1}) x10^{-2}	Sodium azide (s^{-1}) x10^{-2}
Pure Cimetidine peak degradation	9.9	9.2	3.9
Pure FFA peak degradation	4.9	4.7	2.8
Cimetidine peak degradation in 100 μM FFA	8.4	8.1	4.4
FFA peak degradation in 100 μM of cimetidine	4.3	4.7	2.5

Table S4. Stepwise calculation of the steady-state concentration of singlet oxygen ($^1O_2$) based on the observed rate constant (k_{obs}) and the reaction rate constant (k_r)

Parameter	Value
k_{obs} (obtained from the slope of the ln[FFA] vs time graph (Figure S5))	3.3 x 10^{-3} s^{-1}
k_r (reaction rate constant)	1.0 x 10^{8} $M^{-1}s^{-1}$ (ref [3])
$[^1O_2]_{ss}$	3.3 x 10^{-11} M ($moldm^{-3}$)

Table S5. Effect of PS coating on red light transmission through lightguide surfaces. Intensity at 637 nm from the red LED transmitted through the lightguides including emission from all surfaces including the sidewalls and end (No cap) as well as only the sidewalls (Capped) for both smooth and roughened lightguides with and without PS. Intensity measurements were taken by at least three different freshly coated/uncoated lightguides. Measuring at two different integration times (1000 and 2000 ms) demonstrates the reproducibility of the measurements

	Integrating time: 2000 ms				Integrating time: 1000 ms			
	Light intensity (No cap)	% light absorbance	Light intensity (Capped)	% Drop of light intensity upon capping	Light intensity (No cap)	% light absorbance	Light intensity (Capped)	% Drop of light intensity upon capping
Smooth lightguides-No PS	41969 ± 544	**28.9** ± 6.0	2523 ± 158	**94.0** ± 0.4	20532 ± 435	**28.7** ± 3.8	1225 ± 249	**94.0** ± 1.3
Smooth lightguides - With PS	29834 ± 2191		2521 ± 204	**91.6** ± 0.5	14638 ± 925		1076 ± 171	**92.6** ± 1.4
Roughened lightguides - No PS	40110 ± 270	**25.2** ± 1.1	38065 ± 338	**5.1** ± 0.6	20290 ± 121	**25.1** ± 5.2	19136 ± 392	**5.7** ± 1.5
Roughened lightguides - With PS	29998 ± 705		28595 ± 496	**4.7** ± 1.5	15192 ± 1015		14371 ± 929	**5.4** ± 0.4

Table S6. Effect of PS coating on green light transmission through lightguide surfaces. Intensity at 509 nm from the green LED transmitted through the lightguides including emission from all surfaces including the sidewalls and end (No cap) as well as only the sidewalls (Capped) for both smooth and roughened lightguides with and without PS. Intensity measurements were taken by at least three different freshly coated/uncoated lightguides

Lightguide surface	Light intensity (No cap)	% decrease of light intensity	Light intensity (Capped)	% decrease of light intensity	% Drop of light intensity upon capping
Smooth -No PS	36477 ± 581	**5.4** ± 4.0	3722 ± 159	**7.7** ± 3.5	**89.8** ± 0.5
Smooth -With PS	34497± 1333		3435 ± 215		**90.0** ± 0.3
Roughened -No PS	36883± 541	**2.3** ± 4.1	34789 ± 1003	**3.2** ± 4.6	**5.7** ± 1.5
Roughened -With PS	36046± 1081		33663 ± 780		**6.6** ± 1.4

Table S7. Comparison of the intensities of the maximum wavelengths of red and green LED emission coupled to the PS coated lightguides with no cap. The standard deviation represents at least three different measurements

	Red LED - % intensity drop	Green LED -% intensity drop
Smooth lightguides - No cap - With PS	28.9 ± 6.0	5.4 ± 4.0
Roughened lightguides -No cap – With PS	25.2 ± 1.1	2.3 ± 4.1

Table S8. Diffusion coefficient calculation of UA molecule vs *E.coli* bacterium in water

Parameter	Symbol	UA	*E.coli*
Boltzmann Constant	$K\beta$		1.38×10^{-23} (Kg.m^2.s^{-2}.K^{-1})
Temperature	T		300 K
Pi constant	π		3.14
Viscosity of water at 300 K	η		8.57×10^{-4} kg.m^{-1}.s^{-1}
Radius / Hydrodynamic radius	r		5.00×10^{-7} m
**Diffusion coefficient**	D	6.70×10^{-2} m^{2}s^{-1} (ref [4])	5.13×10^{-13} m^{2}s^{-1}
Time	t	7200 s	7200 s
Root mean square displacement	X^2	9.65×10^{-6} m^2	7.38×10^{-9} m^2
Diffusion Length after 2 hours	X	3.11×10^{-3} m	8.59×10^{-5} m
**Distance traveled after 2 hours**		3.1×10^3 µm	85.93 µm

Table S9. Estimating the number of $^1O_2$ collisions required to kill *E. coli* bacterium (see section 13 for more information)

Parameter	Mammalian fibrosarcoma cell[2]	*E. coli* bacterium
Typical diameter	~10 - 20 µm	~1 µm
Assumed diameter for calculation	10 µm	1 µm
Relative cell volume (assuming a spherical shape)	1	1/1000 of mammalian cell
Singlet oxygen molecules required to reduce survival to 1/e	4.6×10^7	5×10^4

References

(1) Aebisher, D.; Bartusik-Aebisher, D.; Belh, S. J.; Ghosh, G.; Durantini, A. M.; Liu, Y.; Xu, Q.; Lyons, A. M.; Greer, A. Superhydrophobic Surfaces as a Source of Airborne Singlet Oxygen through Free Space for Photodynamic Therapy. *ACS Appl. Bio Mater.* **2020**, *3* (4), 2370–2377. https://doi.org/10.1021/acsabm.0c00114.

(2) Zhu, T. C.; Kim, M. M.; Liang, X.; Finlay, J. C.; Busch, T. M. In-Vivo Singlet Oxygen Threshold Doses for PDT. *Photonics Lasers Med.* **2015**, *4* (1). https://doi.org/10.1515/plm-2014-0037.

(3) Appiani, E.; Ossola, R.; Latch, D. E.; Erickson, P. R.; McNeill, K. Aqueous Singlet Oxygen Reaction Kinetics of Furfuryl Alcohol: Effect of Temperature, pH, and Salt Content. *Environ. Sci. Process. Impacts* **2017**, *19* (4), 507–516. https://doi.org/10.1039/C6EM00646A.

(4) Parthasarathy, P.; Vivekanandan, S. Investigation on Uric Acid Biosensor Model for Enzyme Layer Thickness for the Application of Arthritis Disease Diagnosis. *Health Inf. Sci. Syst.* **2018**, *6* (1), 5. https://doi.org/10.1007/s13755-018-0043-3.
